# Modern and Ancient Genomes Reveal Neolithic Paternal Expansions of Millet and Rice Farmers and Demic Diffusion from China into Mainland Southeast Asia

**DOI:** 10.1002/advs.202515930

**Published:** 2026-01-21

**Authors:** Yunhui Liu, Lintao Luo, Yutong Jiang, Minzhu Zhao, Ting Yang, Zhiyong Wang, Lisiteng Luo, Yuhang Feng, Zihao Zhu, Yuzhu Wang, Limei Zhang, Bofeng Zhu, Chao Liu, Renkuan Tang, Mengge Wang, Guanglin He

**Affiliations:** ^1^ Department of Forensic Medicine College of Basic Medicine Chongqing Medical University Chongqing China; ^2^ Institute of Rare Diseases West China Hospital of Sichuan University Sichuan University Chengdu China; ^3^ Center for Archaeological Science Sichuan University Chengdu China; ^4^ Western Forensic Medicine Center of China | Forensic Precision Identification Research and Translation Center College of Basic Medicine Chongqing Medical University Chongqing China; ^5^ Chongqing Key Laboratory of Forensic Science | Chongqing Engineering Research Center of Criminal Investigation Technology College of Basic Medicine Chongqing Medical University Chongqing China; ^6^ School of Forensic Medicine Kunming Medical University Kunming China; ^7^ Guangzhou Key Laboratory of Forensic Multi‐Omics for Precision Identification School of Forensic Medicine Southern Medical University Guangzhou China; ^8^ Microbiome Medicine Center Department of Laboratory Medicine Zhujiang Hospital Southern Medical University Guangzhou China; ^9^ State Key Laboratory of Bioactive Molecules and Druggability Assessment Guangdong Basic Research Center of Excellence for Natural Bioactive Molecules and Discovery of Innovative Drugs Jinan University Guangzhou China

**Keywords:** demic diffusion, founding lineages, genetic structure, Neolithic farmers, Y‐chromosome

## Abstract

The genomic formation and diversity of underrepresented southern Chinese and Southeast Asian populations have long fascinated researchers, particularly regarding Late Paleolithic to Holocene expansions and early paternal interactions. Here, we present a large‐scale paternal genomic aggregation dataset of 14435 ancient and present‐day Eurasian individuals, including 584 newly sequenced whole Y‐chromosome genomes. We reconstruct the highest‐resolution Y‐chromosome phylogeny to date for ancient East and Southeast Asian populations based on our fully resolved modern eastern Eurasian maximum‐likelihood phylogenetic framework. We identify 138 paternal lineages that diversify during the Neolithic in a time‐calibrated phylogeny and find that 17 dominant lineages are shared between China and Mainland Southeast Asia (MSEA), with a marked expansion beginning ca. 5 kya and peaking between 3.5 and 3 kya. While northern and southern Han populations show minimal paternal differentiation, southern ethnolinguistic minorities exhibit clear substructures, in which coastal groups align with Tai‐Kadai speakers, southwestern groups with Hmong‐Mien speakers, and highland groups with Tibeto‐Burman speakers. Our findings support a demic diffusion model of Neolithic farming and Han culture, highlighting the significant paternal contributions of ancient millet farmers and their Han descendants to the genetic landscape of southern China and MSEA, with subsequent enrichment from rice farmer‐mediated expansions.

## Graphical abstract

This study clarifies the genetic patterns of paternal lineages across East Asia and Mainland Southeast Asia. Han populations are relatively homogeneous, whereas southern ethnolinguistic minorities display regional structures. Shared Y‐chromosome lineages indicate Neolithic expansions and extensive north‐south gene flow, supporting demic diffusion associated with millet‐ and rice‐farming dispersals that shaped the paternal genetic landscape of southern China and Southeast Asia.

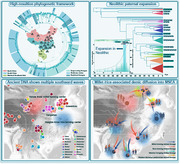

## 1 Introduction

Ancient DNA (aDNA) from spatiotemporally different human groups and large‐scale genomic data from ethnolinguistically diverse populations in the cohort work have greatly enhanced our understanding of migration and admixture processes [1, 2, 3]. Archaeological and genetic evidence support the hypothesis that millet‐farming communities originating from the Yellow River basin expanded around 6000 years ago, spreading millet agriculture westward into the Qinghai‐Xizang Plateau and contributing to the divergence of Sino‐Tibetan‐speaking populations and their associated languages [3, 4, 5, 6, 7]. Autosomal aDNA from the Yangtze River basin and Mainland Southeast Asia (MSEA) further highlights the genetic links between southern Chinese farmers and the Neolithic‐to‐Iron Age populations of MSEA [7, 8, 9, 10, 11]. However, despite the substantial contribution confirmed by autosomal studies, Y‐chromosome data remain limited, constraining insights into male‐driven evolutionary histories.

East Asia (EA) and MSEA are home to a vast cultural and linguistic diversity, including the world's largest Han Chinese population alongside numerous ethnolinguistically different groups. This diversity is shaped by large‐scale migrations, geographic isolation, and environmental variation, generating distinct genetic patterns and complex interconnections among populations [12, 13, 14]. The Han population, historically rooted in the Huaxia tribes of the Yellow River basin, has expanded over the past two millennia, particularly into the Qinghai‐Xizang Plateau and southern China. During this process, it interacted with indigenous groups such as Tai‐Kadai, Austroasiatic, and Hmong‐Mien speakers [12, 13, 15]. While early interpretations of dialectal and cultural differences between northern and southern Han populations emphasized cultural diffusion [16], the presence of shared Y‐chromosome and mitochondrial DNA (mtDNA) haplotypes suggests significant contributions from demic diffusion [13, 17, 18]. Recent studies, including those employing the Weakly‐Differentiated Multi‐source Admixture model by Wang et al., have clarified the role of complex paternal founding lineages in shaping Han's genetic structure [19]. Nevertheless, whether the paternal expansion of Neolithic East Asian farmers, Han Chinese people, and their Holocene ancestral populations into MSEA is primarily driven by population diffusion or cultural transmission remains unresolved.

Southern China is situated at the intersection of EA and MSEA. It represents a major center of linguistic and cultural diversity, giving rise to at least four prominent language families: Tai‐Kadai, Hmong‐Mien, Austronesian, and Austroasiatic [15, 20]. From a paternal genomic perspective, haplogroups such as O1a‐M119, O2a2a1a2‐M7, and O1b1a1a‐M95 have been linked to Austronesian as well as Tai‐Kadai, Hmong‐Mien, and Austroasiatic speakers, respectively [21, 22, 23, 24]. Research on the genetic diversity of southern China has primarily focused on Han populations [18, 25, 26, 27] or specific minority groups, revealing high genetic diversity and complex admixture histories [28, 29, 30, 31, 32]. Advancements in aDNA technologies have revolutionized population genetic research, providing new insights into human genetic history and evolutionary processes [1, 3, 33, 34, 35]. Ancient genomes from the Gaoshan and Haimenkou sites indicate that Neolithic millet‐farming communities from the Yellow River basin migrated southwestward into regions such as Sichuan and Yunnan [36]. Similarly, genomic analysis of ancient remains from Guizhou has further supported the demic diffusion model of Han culture in southwestern China [37]. Studies of ancient individuals from Vietnam have revealed a mixture of distinct ancestors. These ancestors include southern Chinese agriculturalists and eastern Eurasian hunter‐gatherers, highlighting the deep genetic connections between populations from EA and MSEA [8, 9]. Such studies, in conjunction with archaeological evidence, support the hypothesis that Neolithic farmers from southern China migrated into MSEA [8, 9]. These movements contributed significantly to the genetic composition of modern populations in the region. However, the extent to which the southward migration of Neolithic farmers shaped the paternal genetic makeup of southern Chinese ethnic minorities and populations from MSEA remains unclear.

Current Y‐chromosome sequencing data from China and MSEA are limited, predominantly focusing on Han populations [19, 38, 39, 40] or low‐coverage data [41, 42, 43, 44], underscoring the need for more extensive sampling across East Asian multi‐ethnic populations. Recent advancements in sequencing technologies and computational biology have enabled the generation of comprehensive genomic datasets, offering more precise reconstructions of the deep human evolutionary events based on the variation spectrum documented in Y‐chromosomes. Large‐scale genomic resources, such as the All of Us Research Program [45], UK Biobank [46], and 1000 Genomes Project [47]. Human Genome Diversity Project [48], and 10K Chinese People Genomic Diversity Project [49], demonstrate that populations with diverse ethnolinguistic backgrounds exhibit distinct genetic architectures. The Y‐chromosome, which is largely unaffected by recombination, offers a powerful tool for tracing the origins and migration patterns of paternal lineages [35, 38, 50, 51, 52, 53]. Recent improvements in genome assembly and variant detection have overcome past challenges in studying Y‐chromosome variation, facilitating the construction of robust, time‐calibrated phylogenies [54, 55, 56, 57]. Nevertheless, the diversity of human Y‐chromosomes has been underrepresented, and their contributions to human evolutionary research have been limited. To address these gaps, we compiled a comprehensive Y‐chromosome dataset comprising 13355 modern and 1080 aDNA sequences (Tables S1 and S2). Our findings of phylogeographical signatures, genetic diversity, and fine‐scale paternal structures provide new insights into the genetic relatedness and differentiation between populations from EA and MSEA, as well as the paternal genetic connections between ancient Chinese farmers and contemporary and ancient populations in MSEA.

## 2 Results

### 2.1 A Comprehensive and High‐Resolution Y‐Chromosome Phylogeny with Robust Dates

**FIGURE 1:**
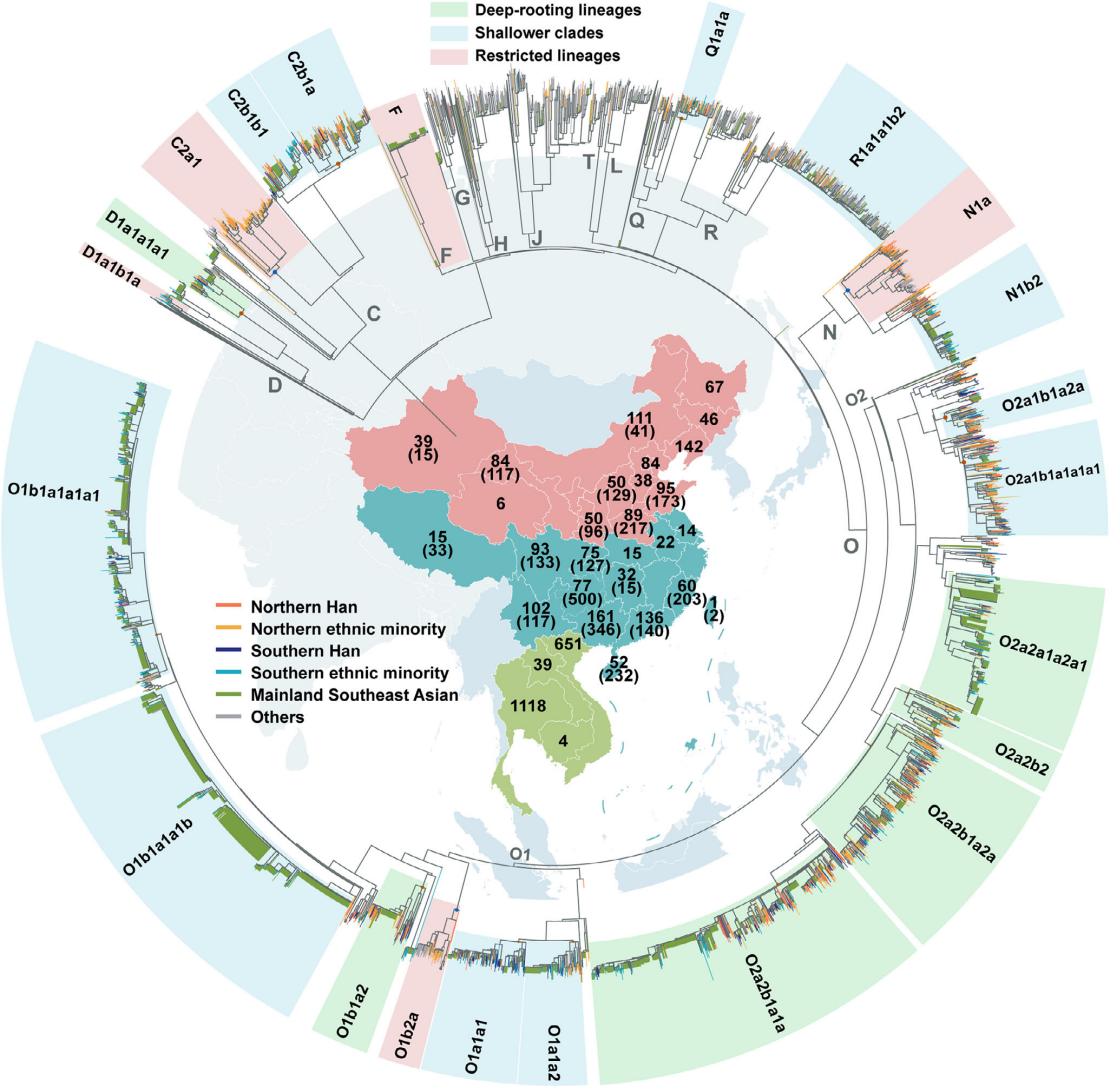
Geographic distribution and phylogenetic topology of ethnolinguistically diverse eastern Eurasian populations. Maximum likelihood (ML) tree based on 5363 Y‐chromosome sequences from China and Mainland Southeast Asia (MSEA), placed in a global paternal lineage context. The inset map highlights the studied region, with the Qinling‐Huaihe Line (between the Qinling Mountains and Huaihe River) used to delineate northern and southern China. Regional colors correspond to distinct geographic zones: vermilion for northern China, blue for southern China, and green for MSEA. Numbers indicate sample sizes for each province or country, with values outside parentheses representing whole‐genome sequencing data and those in parentheses representing genotyping data. Other regions of East Asia (EA) and Southeast Asia are also highlighted on the map for geographic context. Branches of the ML tree are color‐coded to match the geographic regions shown on the map: red and orange hues for northern China, dark and light blues for southern China, green for MSEA, and gray for other regions. Boxed clades indicate labeled haplogroups and their downstream branches. Light blue boxes highlight shallower clades shared between China and MSEA; light green boxes denote deep‐rooting lineages shared between these regions; light red boxes indicate haplogroups with more geographically restricted distributions. Clades A, B, E, and other lineages predominantly found outside the studied region were collapsed. One sample belonging to haplogroup NO and two samples exhibiting excessively long branch lengths were removed. The root was truncated to improve visualization. For detailed sample information, see Figure S1 and Table S1.

**FIGURE 2:**
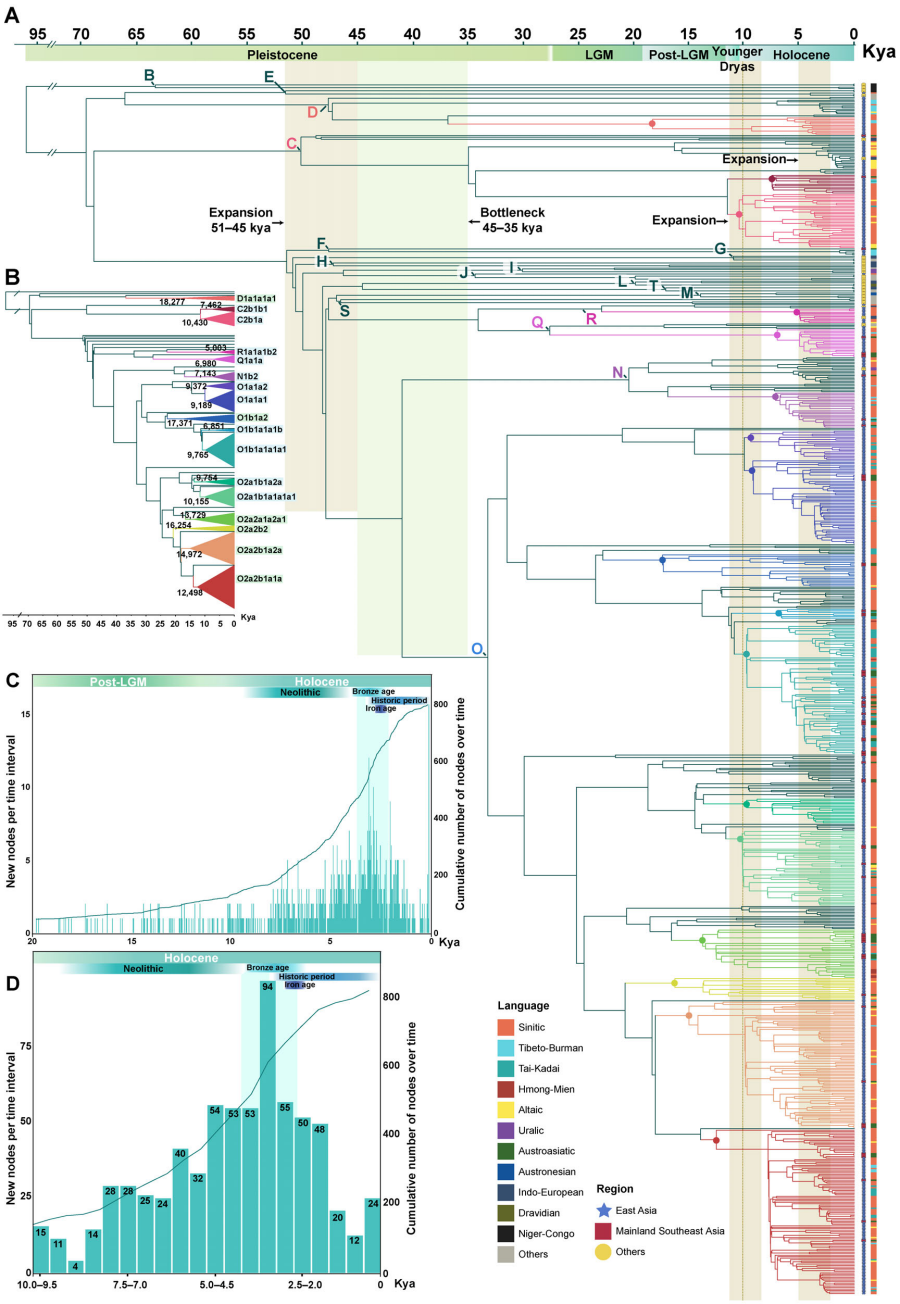
Paternal lineages shared between China and MSEA with robust time estimates. (A) Phylogeny based on 823 Y‐chromosome sequences. Shapes following the terminal nodes indicate geographic origin: blue stars for EA, red squares for MSEA, and yellow circles for other regions. A strip on the right side of the tree denotes the language family. Boxed regions within the tree highlight periods of notable expansions or bottlenecks. Seventeen lineages shared between China and MSEA (identified in Figure 1) are color‐coded along the branches. (B) Collapsed overview of the tree in (A). Box background colors are the same as in Figure 1. The time to the most recent common ancestor (TMRCA) for each lineage is annotated. Other clades are simplified. (C) Number of new and cumulative branching events inferred from the 823 sequences over the past 20000 years, using a generation time of 29 years (commonly used in autosomal studies). (D) Same as (C), focusing on the past 10000 years, shown in 500‐year intervals.

To construct a high‐resolution landscape of paternal lineages across China and MSEA, we compiled a global dataset of 5366 whole Y‐chromosome sequences and constructed a maximum‐likelihood (ML) tree to trace the paternal demographic history of global populations (Figure 1; Figure S1 and Table S1). Of these, 3618 sequences covered approximately 8.4 Mb of high‐confidence genomic regions that were extracted, including 584 newly reported samples from diverse ethnic groups across 14 provinces in China, supplemented by data from public repositories and previous studies [35, 58, 59, 60]. Notably, 1748 sequences from MSEA were used to explore the genetic connections between China and MSEA [44, 61, 62], while exhibiting lower coverage (ca. 2.3 Mb). The reconstructed ML‐based phylogenetic tree and Bayesian evolutionary analysis sampling tree (BEAST) identified 138 lineages that diversified during the Neolithic and 17 paternal lineages shared by China and MSEA, which fall within the downstream clades of D‐CTS3946, C‐M130, Q‐M242, R‐M207, N‐M231, and O‐M175 (Figures 1 and 2). These clades displayed varying levels of divergence: six had long branches with deep‐rooting nodes, while the remaining eleven showed shallower clades (Figure 1). Time to the most recent common ancestor (TMRCA) estimates, derived using BEAST (Figure 2A,B and Table 1), corroborated this observation. Our analysis revealed a major diversification event around 51–45 thousand years ago (kya), followed by a significant bottleneck (ca. 10000 years, 45–35 kya) across most lineages (Figure 2A). Subsequently, haplogroup O demonstrated a marked increase in diversity from the Upper Pleistocene to the Holocene. In contrast, other shared lineages (C, D, N, Q, and R) remained relatively unbranched until the Neolithic. The newly emerged and cumulative nodes represent the number of coalescent events. On the 20000‐year timescale, a noticeable increase in node counts began around the onset of the Neolithic (Figure 2C), consistent with the hypothesis that the rise of Neolithic agriculture drove population growth [63]. Within the Holocene, the rate of node accumulation accelerated markedly around five kya (>50 new nodes per time interval), reaching a peak between 3.5 and 3 kya with 94 newly inferred coalescent events (Figure 2D). These findings suggest that most paternal genetic exchanges between China and MSEA occurred during the Neolithic, with haplogroup O emerging as the dominant lineage in the paternal gene pools of China and MSEA.

**TABLE 1:** TMRCA estimates for 17 haplogroups shared between China and MSEA, inferred using BEAST.

Lineage	Number	Branch	Mean TMRCA [Year Ago]	95% HPD Interval
C2b1a	37	shallower	10430	9401–11416
C2b1b1	13	shallower	7462	6810–8272
Q1a1a	19	shallower	6980	5956–7837
R1a1a1b2	10	shallower	5003	4117–5756
N1b2	24	shallower	7143	6382–7988
O2a1b1a2a	17	shallower	9754	8688–10988
O2a1b1a1a1a1	51	shallower	10155	9347–11061
O1a1a2	20	shallower	9372	8416–10331
O1a1a1	57	shallower	9189	8155–10037
O1b1a1a1b	7	shallower	6851	5761–7737
O1b1a1a1a1	88	shallower	9765	8900–10603
D1a1a1a1	12	deep	18277	16060–20524
O2a2b1a1a	112	deep	12498	11120–14002
O2a2a1a2a1	33	deep	13729	12424–14922
O2a2b2	15	deep	16254	14714–17871
O2a2b1a2a	86	deep	14972	13614–16349
O1b1a2	22	deep	17371	15772–19009

The ML tree topology and the geographic distribution of terminal nodes further provide insights into these paternal phylogenetic relationships and migration patterns. Using pathPhynder‐based phylogenetic placement [64], we positioned the MSEA sequences onto the highest‐resolution branches of the Eurasian Y‐chromosome tree (Figure 1). This likelihood‐based approach enabled a high‐resolution integrative phylogeny of China and MSEA, avoiding potential information loss that may occur when phylogeny reconstruction relies solely on the shared 2 Mb region of modern human sequences (Methods), while also maximizing the utility of the currently available MSEA data. We observed apparent lineage radiations across China and MSEA for most shared clades. A general north‐to‐south migration pattern from China emerged in 17 shared clades, with multiple lineages originating in China and dispersing into MSEA at various time points. Haplogroup O2a2b1a1a, a known lineage of the Yangshao‐related millet farmers, is now broadly distributed across China and MSEA and has numerous downstream subclades (e.g., O2a2b1a1a1a to O2a2b1a1a1g; Figure S2 and Table S1), suggesting an early and rapid expansion. The upstream clustering position of northern Han and minority populations in many subclades further supports the hypothesis that these southward dispersals significantly shaped the paternal genetic structure of southern China and MSEA. Additionally, several clades showed regionally restricted distributions (Figure 1). D1a1b1a was confined to Tibeto‐Burman groups in southwestern China and a few northern populations, while C2a1, O1b2a, and N1a were primarily confined to northern China. Interestingly, haplogroup F, though rare, was more prevalent in MSEA. Expanding the sampling in these regions may yield critical insights into the evolutionary trajectories of these poorly understood lineages.

### 2.2 Paternal Genetic Structure and Contributing Forces of Ethnolinguistically Diverse Chinese Populations

#### 2.2.1 Genetic Landscape and Diversity

**FIGURE 3:**
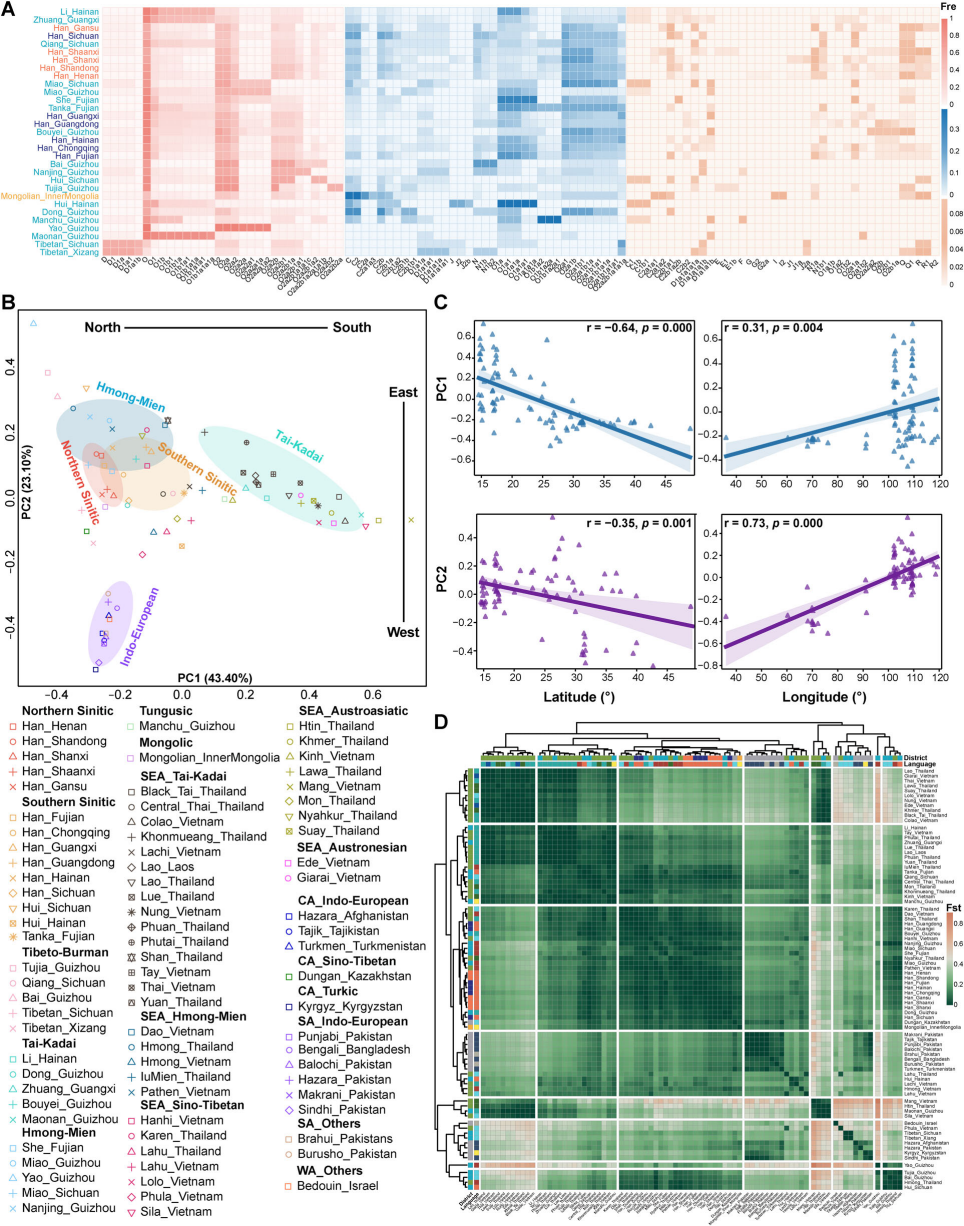
Haplogroup frequencies and population structure. (A) Heatmap of haplogroup frequencies in newly reported genotyping populations. Population labels are color‐coded according to the geographic regions defined in Figure 1. The intensity of the heatmap color indicates frequency levels, with darker shades representing higher frequencies. Fre: frequency. (B) Principal component analysis (PCA) of the newly generated genotyping data combined with reference populations. Colored ellipses highlight populations affiliated with different language families. SEA: Southeast Asia; CA: Central Asia; SA: South Asia; WA: West Asia. (C) Pearson correlation between PC values from (B) and geographic coordinates (latitude and longitude) of the corresponding populations (n = 82), and *p* < 0.05 was considered significant. (D) Pairwise genetic distance (*F*st) analysis among the genotyped and reference populations. Darker green indicates lower *F*st values (closer genetic affinity), while deeper orange represents higher *F*st values (greater genetic divergence). Language family and regional color codes follow the same convention as in Figure 2.

The ML tree and BEAST estimates offered preliminary insights into genetic similarities, shared ancestral coalescent times, and the potential effects of southward migrations between China and MSEA. However, due to the limited sample sizes, the fine‐scale paternal genetic structure and the forces shaping this genetic legacy remain poorly understood. We expanded our sample size to address this gap by genotyping an additional 7877 individuals from ethnolinguistically diverse groups and targeted founding lineages using a cost‐effective, high‐resolution microarray approach (Figure 1; Table S1). This large‐scale effort aims to clarify the genetic impacts of north‐to‐south dispersals on the paternal landscape of southern China and MSEA. The ethnic minority populations sampled were predominantly from South China, which possessed rich linguistic and ethnic diversity (Figure S3). Owing to its intermediate geographic position between EA and MSEA, South China has long served as a pivotal region for population interactions, cultural diffusion, and genetic exchanges across these areas. Despite its potential genetic complexity, it remains underrepresented in human genomic studies. We identified the major haplogroups as O‐M175 (75.2%), C‐M130 (8.6%), N‐M231 (5.9%), D‐CTS3946 (4.2%), Q‐M242 (2.5%), and R‐M207 (1.6%) (Figure S3D). Notably, the distribution of these haplogroups varied significantly across populations (Figure 3A; Table S3). After excluding groups with fewer than 20 individuals, we observed that haplogroup O was predominantly represented by O2‐M122, with a mean frequency of 47.17% across all 31 ethnolinguistically diverse populations. This lineage showed a higher prevalence in the six northern populations (mean: 50.68%) compared to the remaining 25 southern populations (mean: 46.33%) (Table S3). The C2‐M217 and N1‐CTS3750 lineages were more common in northern populations but also appeared in several southern minorities, while O1‐F265 was notably enriched in southern populations. D1‐M174 was mainly confined to Tibetan groups, with C1‐F3393, Q‐M242, and R‐M207 occurring sporadically, primarily in northern regions. Additionally, specific rare subclades, including J2‐M172, I2‐M438, and F‐M89, reached significant frequencies within particular groups (Table S3). These geographically distinct distributions suggest varying paternal origins or complex admixture histories.

Comparisons with Southeast Asian reference populations revealed that Chinese groups shared close genetic affinities and broadly similar haplogroup profiles with populations in MSEA (Figure S4A). Despite this overall similarity, we also observed differentiated patterns via Mann‐Whitney U test and Welch's t‐test, in which O1a and O2a were more prevalent in Chinese populations, while O1b was significantly enriched in MSEA groups (*p* < 0.05) (Figure S4B). Meanwhile, statistical tests among linguistically related populations revealed genetic affinities among these groups. No significant differences emerged between Hmong‐Mien and Tai‐Kadai speakers across China and MSEA, supporting a shared origin or frequent gene flow (Figure S4C,D). In contrast, Sino‐Tibetan speakers showed variation in O1a and O2a, contributing to regional population genetic divergence (Figure S4E). We also detected traces of Central and South Asian haplogroups in geographically restricted northwestern Chinese populations, suggesting limited eastward gene flow (Figure S4A). To assess paternal genetic diversity, we calculated haplogroup diversity (HGD), haplotype diversity (HD), mean pairwise differences (Pi), Theta(S), Tajima's *D*, and Fu's FS (Table S4 and Figure S5). Han populations, particularly in the north, exhibited the highest genetic diversity. Among minority groups, southwestern populations showed polarized patterns: some groups, such as the Qiang and Dong, had high diversity, while others exhibited reduced variation. Frontier minorities, including the Zhuang, She, and Mongolians, displayed relatively high diversity. These trends were consistent across Pi and Theta(S), reflecting a large effective population size and rich paternal genetic diversity in Han groups. Several southern populations, like Zhuang and Tujia, showed significantly negative Tajima's *D* and Fu's FS values, indicating recent paternal expansions, particularly in coastal and southwestern regions. Northern Han and certain southwestern groups likely retain ancient genetic lineages shaped by complex admixture histories.

#### 2.2.2 Multiple Socio‐Cultural Factors Contributed to Geography‐ and Language‐Related Genetic Structures

To further investigate the genetic substructure of East and Southeast Asian groups and possible influencing factors that contributed to it, we performed principal component analysis (PCA) based on haplogroup frequencies. PC1 and PC2 explained 61.52% and 18.46% of the variance (Figure S6A), revealing distinct north‐south differentiation along with PC2. A negative correlation between PC2 and latitude further emphasized this spatial pattern (r = −0.39, *p* = 0.034) (Figure S6B). Northern and southern Han populations clustered closely, while southern ethnic minorities displayed greater genetic distinctiveness. Linguistic distinctions were also evident: northern Sinitic groups showed genetic homogeneity, while southern Sinitic groups exhibited greater heterogeneity. Some Tibeto‐Burman and Hmong‐Mien groups, e.g., Qiang, Miao, and She, were genetically close to Han populations, whereas others (e.g., Dong, Yao, Maonan) exhibited notable divergence. Despite relative isolation in some groups, substantial genetic links were observed between the Han and certain ethnic groups. At the broader Eurasian scale (Figure 3B), PCA revealed both a north‐south cline along PC1 and a west‐east cline along PC2, consistent with correlation analyses (Figure 3C). Populations from EA and MSEA, especially Tai‐Kadai, Hmong‐Mien, and Sino‐Tibetan speakers, formed a close genetic cluster, highlighting their genetic affinity. The Hui populations exhibited regional genetic variations, suggesting multiple origins or differentiated admixture histories. The Tanka people were displaced far from southern Sinitic speakers, likely due to their isolated maritime lifestyle [65]. Tibetans clustered with northern Han and Dungan populations, indicating a Neolithic common origin or recent genetic exchange. The Yao in Guizhou maintained a distinct genetic profile, consistent with their historically documented geographical and cultural isolation. Overall, the paternal genetic structure of EA and MSEA may be shaped by a combination of shared ethnic origins, cultural similarities, multidirectional migrations, and geographic isolation.

Pairwise genetic distance (*F*st) analysis revealed notable patterns of genetic affinity and divergence. Northern and southern Han populations showed close genetic relationships (<0.0697), with low *F*st values observed between Henan and Chongqing (0.0122) and between Shaanxi and Chongqing (0.0129) (Figure S6C and Table S5). Genetic affinities were also evident between specific Han populations and ethnic minorities, such as the Qiang and Guangdong Han (0.0209) and between the Fujian Tanka and Hainan Han (0.0173). In contrast, populations like the Tibetan (0.1921–0.5920 for Xizang, 0.1557–0.5757 for Sichuan), Yao (0.1023–0.7078), and Maonan (0.0956–0.7078) exhibited substantial divergence from other reference groups. These patterns were further corroborated by an unweighted pair‐group method with arithmetic mean (UPGMA) phylogenetic tree (Figure S6D). When MSEA populations were included, distinct genetic ties emerged among Tai‐Kadai (0.0251–0.3186), Hmong‐Mien (0.0245–0.4460), and Sino‐Tibetan speakers (0.0177–0.6101) (Figure 3D; Table S6). Regional clustering within the Hui populations was evident, with those from Hainan aligning with Tanka and Mon, while those from Sichuan grouped with Han and Bouyei. Tibetans clustered with Dungan and northern Han, while the Maonan grouped with Lawa and Tay.

To assess the factors driving the observed genetic diversity, we conducted an analysis of molecular variance (AMOVA). Ethnicity emerged as the primary determinant of genetic variance among Chinese populations (group 4, 8.99%), followed by altitude (group 8, 6.2%), language (group 9, 4.15%), and the north‐south divide (group 7, 4.03%; group 6, 1.52%; group 2, 1.04%) (Table S7). Minimal genetic differentiation was observed between northern and southern Han populations (group 3, 0.97%), reflecting their high internal homogeneity. In contrast, ethnic minorities exhibited considerable genetic variance, with altitude playing a more significant role in shaping their genetic structure. Notably, the genetic variance between northern and southern minorities was substantial (group 2, 7.53%). When incorporating Eurasian references (Table 2), genetic differences between East and Southeast Asian populations were modest (group 4, 5.72%), with even more minor differences between populations from southern EA and MSEA (group 6, 4.33%). Among linguistically diverse groups, the Tai‐Kadai (group 7, 6.8%) exhibited the lowest genetic differentiation, while the Hmong‐Mien (group 8, 9.67%) and Sino‐Tibetan (group 9, 10.04%) groups showed higher levels of genetic differentiation. These findings reinforced the PCA and *F*st results, emphasizing the genetic homogeneity between southern East Asian and Southeast Asian populations.

**TABLE 2:** AMOVA results stratified by ethnolinguistic groups across Eurasian populations.

Groupings	Number of Populations	Number of Groups	Percentage of Variation		
			Among‐Groups	Among Populations Within Groups	Within‐Populations
**1. All**	82	1	−	16.05	83.95
**2. East Asia vs Southeast Asia vs South Asia vs Central Asia vs West Asia**	82	5	9.14	10.1	80.76
**3. (East Asia and Southeast Asia) vs (South Asia, Central Asia and West Asia)**	82	2	13.56	11.88	74.55
**4. East Asia vs Southeast Asia**	68	2	5.72	10.84	83.44
**5. Northern East Asia vs Southeast Asia**	43	2	9.45	10.06	80.49
**6. Southern East Asia vs Southeast Asia**	62	2	4.33	12.88	82.79
**7. Tai‐Kadai (East Asia and Southeast Asia)** ^a^	20 (20)	2 (1)	−0.87* (−)	7.25 (6.8)	93.62 (93.2)
**8. Hmong‐Mien (East Asia and Southeast Asia)** ^a^	20 (20)	2 (1)	−0.12* (−)	9.74 (9.67)	90.38 (90.33)
**9. Sino‐Tibetan (East Asia and Southeast Asia)** ^a^	20 (20)	2 (1)	7.79 (−)	7.49 (10.04)	84.73 (89.96)

^a^ In groups 7, 8, and 9, the values in parentheses show the analysis grouping linguistic families into a single category. Asterisks indicate that the value is not significant based on permutation tests (*p* > 0.05).

#### 2.2.3 Genetic Architecture and Founding Lineages of East Asians

**FIGURE 4:**
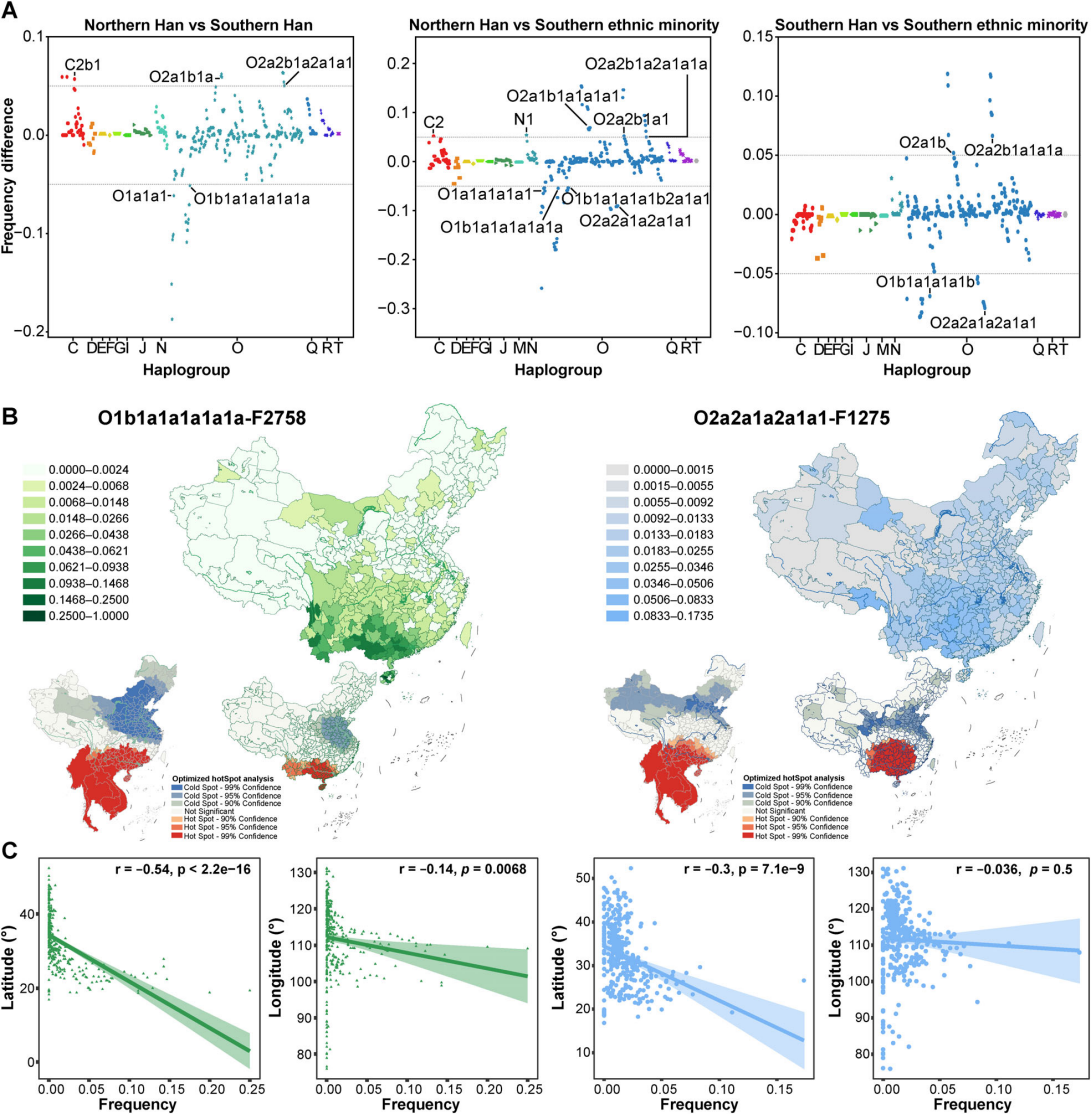
Highly differentiated haplogroups (HDHs) and origins of founder lineages. (A) HDHs identified between Han and ethnic minority populations, with only terminal subclades labeled. A full list of HDHs is available in Table S8. Haplogroup distributions were compared using chi‐square tests and Fisher's exact tests, with *p* < 0.05 considered statistically significant. Sample sizes (numbers of individuals per haplogroup and population) are shown in Table S8. (B) Two founder haplogroups associated with Tai‐Kadai and Hmong‐Mien populations. The upper panel shows haplogroup frequency maps, with darker colors indicating higher frequencies. The lower panel displays an optimized hotspot analysis, where deeper red regions indicate a higher likelihood of origin and expansion centers. (C) Pearson correlation analysis between the frequencies of the two founder haplogroups across prefecture‐level cities (n = 360) and geographic coordinates, with *p* < 0.05 considered significant. Line colors correspond to the haplogroups shown in (B).

Although previous results highlight genetic affinities and distinctions among ethnolinguistically diverse populations, the underlying genetic mechanisms driving these patterns remain unclear. To better understand the genetic differences between northern and southern Han populations and between Han and ethnic minorities, we analyzed haplogroup frequency variations. We identified highly differentiated haplogroups (HDHs) using a differentiation threshold of 0.05 (Methods). Among the HDHs distinguishing northern and southern Han populations, northern lineages, such as C2, N1, and O2a subclades, were more prevalent, while southern Han populations exhibited higher frequencies of downstream O2a1b and O2a2b lineages (e.g., O2a2b1a1a1a) (Figure 4A; Table S8). In contrast, ethnic minorities carried distinct haplogroups, notably O2a2a1a2a1a1 and O1b1a1a1a1b. These HDHs highlight the role of geographic barriers, such as the Qinling‐Huaihe Line, and cultural identity in driving genetic differentiation between Han and minority groups. Further analysis revealed associations between lineage distribution and linguistic groups (Figure S7 and Table S9). Tibeto‐Burman and Sinitic speakers of northern origin exhibited O2a variants, while southern Tai‐Kadai and Hmong‐Mien speakers carried O1b and O1a derivatives. The elevated frequencies of O2a2a1a2a1a1a1 and O2a2b1a1a1c1a1a1a in Hmong‐Mien‐speaking populations likely explain their close affinity with Han populations in PCA. These findings underscore the strong correlation between paternal lineages and linguistic affiliations.

To explore the distribution and evolutionary patterns of these HDHs, we constructed median‐joining (MJ) network diagrams (Figures S8 and S9). The MJ networks revealed star‐like expansions of O1b1a1a1a1a1a in Tai‐Kadai‐speaking populations and O2a2a1a2a1a1 in Hmong‐Mien‐speaking populations (Figure S8). Phylogeographical analysis of key mutations, selecting 1852 samples from O1b1a1a1a1a1a‐F2758 and 3389 from O2a2a1a2a1a1‐F1275 (Table S1), identified these lineages as most prevalent in the southwestern coastal and inland regions, with optimized hotspot analysis confirming these areas as likely origins (Figure 4B). A significant negative correlation with latitude further supports the hypothesis of southern expansion centers of these lineages (Figure 4C). Interestingly, when incorporating lower‐resolution haplogroup data from MSEA (aggregated at the national level), the hotspot extended further into MSEA (Figure 4B). Given the documented genetic turnover in this region involving East Asian ancestry, this pattern may further suggest recent southward migration from southern China into MSEA. These two haplogroups thus emerge as the founding lineages of Tai‐Kadai and Hmong‐Mien groups, respectively.

### 2.3 Highest‐Resolution Y‐Chromosome Phylogenies for Ancient East and Southeast Asian Populations

**FIGURE 5:**
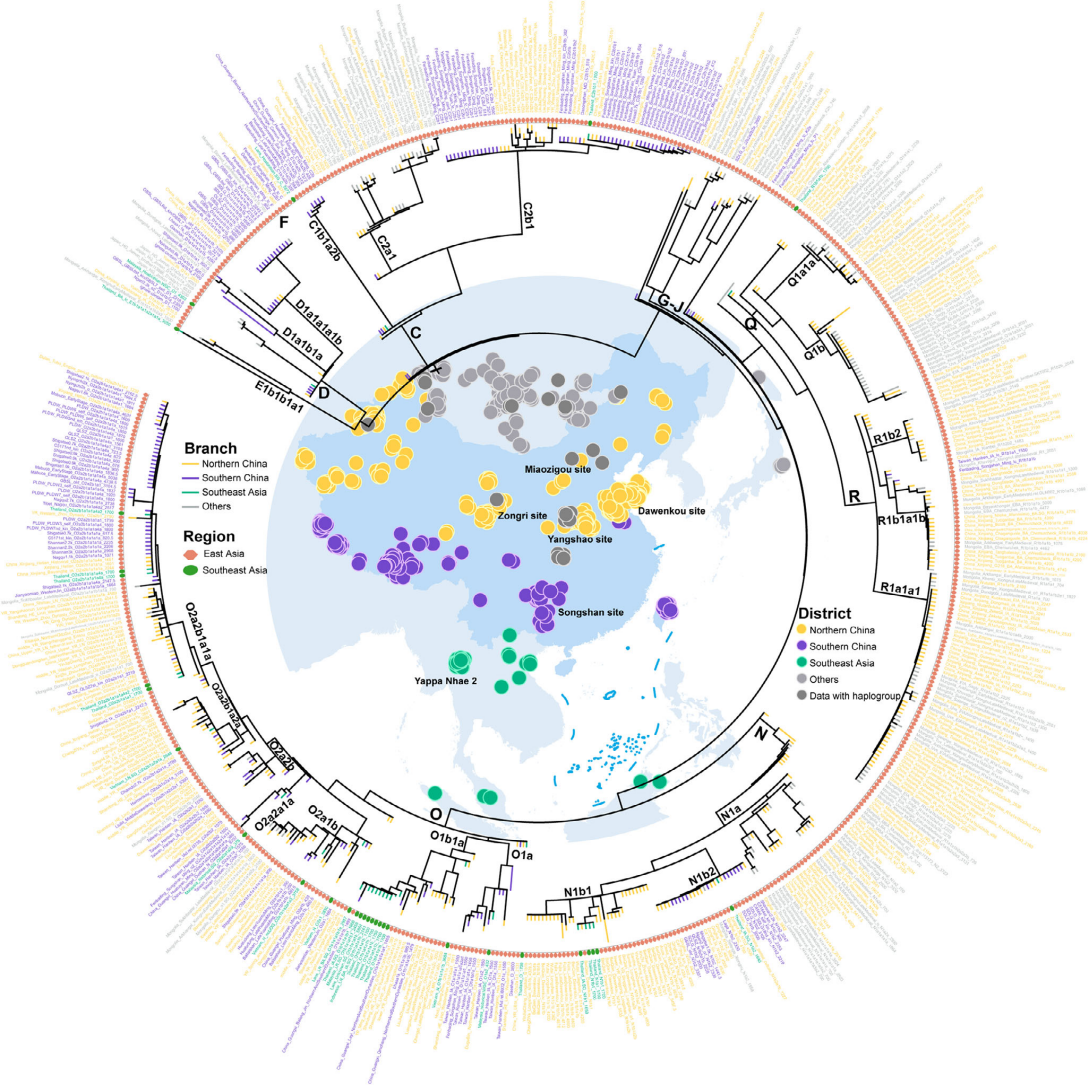
The highest‐resolution Y‐chromosome phylogeny of ancient East and Southeast Asian populations. The inset map displays the geographic origins of the ancient DNA (aDNA) samples. Circle colors on the map correspond to the branch colors in the tree; dark gray circles indicate aDNA for which only haplogroup information is available. Shapes near the terminal nodes of the ML tree indicate regional affiliation: red diamonds represent East Asian samples, while green ovals denote Southeast Asian samples. The color of each sample label in the outer ring matches the corresponding branch color.

**FIGURE 6:**
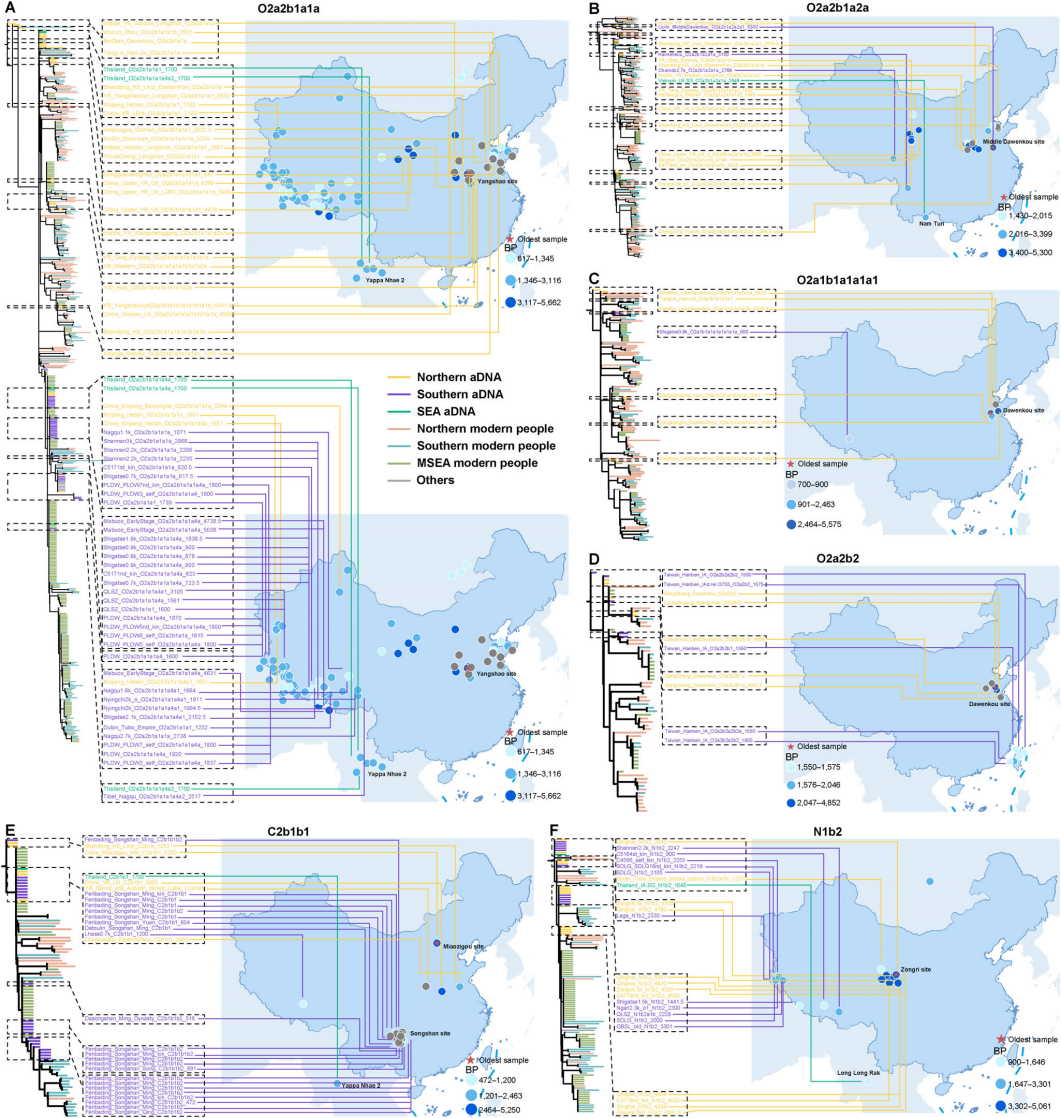
Placement of modern Southeast Asian males (ca. 2.3 Mb coverage) and ancient samples into the ML tree. The tree structure illustrates the phylogenetic topology of the focal haplogroups across ancient and present‐day individuals, while the map shows the geographic locations of aDNA, including those without BAM files but with haplogroup data. Lines connect ancient samples to their respective locations. When available, sample ages (in years BP) are indicated after the haplogroup name. Branch and line colors reflect the regional origin of ancient individuals: yellow for northern individuals, purple for southern individuals, and dark green for Southeast Asian individuals. Branch colors for modern individuals follow the color scheme used in Figure 1, with other modern and ancient samples shown in gray. Each circle on the map represents an ancient individual, with deeper blue shades indicating older ages. Red pentagrams indicate the oldest samples. Major archaeological sites are also marked on the map. (A) O2a2b1a1a. (B) O2a2b1a2a. (C) O2a1b1a1a1a1. (D) O2a2b2. (E) C2b1b1. (F) N1b2.

Genetic differentiation is evident across some East and Southeast Asian populations; however, substantial connections persist between northern and southern Han groups, Han populations, and ethnic minorities, as well as East and Southeast Asian populations, as shown by PCA, *F*st, and AMOVA analyses. In addition, analyses of phylogenetic tree topologies, genetic diversity, and haplogroup frequencies consistently indicate an ancient northern origin for southern Han populations and several minorities, with a notable genetic contribution from southward migrations of southern Chinese to the paternal makeup of MSEA. We mapped the spatiotemporal distributions of shared haplogroups between East and Southeast Asian populations using a single, large‐scale integrative aDNA dataset (Table S2) to test this hypothesis. In addition, using a fully‐resourced modern eastern Eurasian ML‐based phylogenetic framework, we reconstructed the highest‐resolution Y‐chromosome phylogenies to date for ancient East and Southeast Asian populations (Figures 5, 6). The ancient eastern Eurasian Y‐chromosome phylogenetic topology showed a pattern similar to that observed among modern populations, providing direct spatiotemporal connections among modern and ancient East and Southeast Asian populations. Our results identified signals of north‐to‐south migration across multiple founding lineages among ancient individuals or integrated ancient and contemporary MSEA groups. For example, O2a2b1a1a, widely regarded as representative of early farmers from the Yellow River basin, was identified in the Yangshao Village, dated to 5662 years before the present (BP) (Figure 6A). Its offspring subsequently expanded northward into Mongolia, westward into Northwest China, southward into the Qinghai‐Xizang Plateau, and further into Thailand. Other widespread lineages, such as O2a2b1a2a (Figure 6B), initially presented in the Yellow River basin between 5300 and 3250 BP, later reached the Qinghai‐Xizang Plateau and Vietnam (2789–1430 BP). In contrast, haplogroups like O1a, O1b1a1a1, and O2a2a1a2, which are now prevalent in MSEA, exhibit patterns consistent with southward migration from the Yangtze River basin, as evidenced by findings from Liangzhu, Wucheng, and Daxi [22]. Moreover, the earliest instances of O2a1b1a1a1a1 and O2a2b2 were found in Shandong (Figure 6C,D), whereas ancient individuals belonging to the C2b1b1 and N1b2 lineages were predominantly found at the Miaozigou site and the Qinghai‐Xizang Plateau (Figure 6E,F), respectively. These spatiotemporal patterns provide robust evidence for multiple southward migration waves.

**FIGURE 7:**
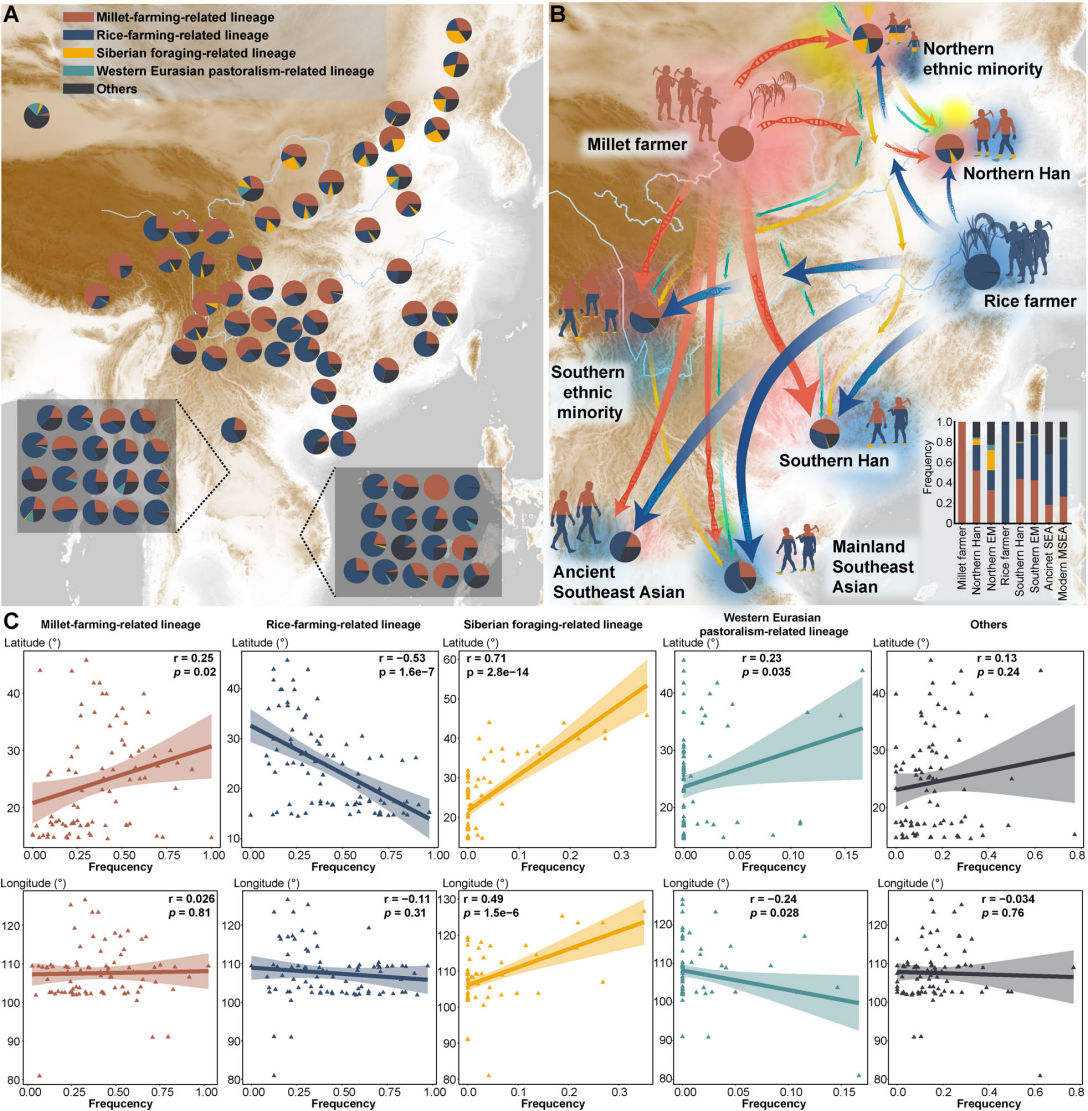
Paternal genetic contributions of ancient agriculturalists to present‐day China and MSEA. (A) Frequencies of subsistence‐related paternal haplogroups across 86 Chinese and MSEA populations. MSEA groups are displayed in boxes. Populations on the lower left are from Thailand; those on the lower right are from Vietnam. (B) Contributions of millet‐ and rice‐farming‐associated haplogroups to ancient and modern meta‐populations. "EM" denotes ethnic minorities. Arrow colors match the haplogroups shown in (A); arrow thickness corresponds to haplogroup frequencies within each meta‐population; arrow paths are schematic and do not represent actual migration routes. Color gradients indicate migration directions, with lighter colors showing origins and darker colors showing destinations. Light blue lines indicate the Yellow and Yangtze Rivers. The inset bar plot shows haplogroup frequencies within each meta‐population. (C) Pearson correlation analysis between the frequencies of subsistence‐related haplogroups across Chinese and MSEA populations (n = 86) and geographic coordinates, with *p* < 0.05 considered significant. Line colors match those in (A). Frequency values in (A) and (B) are provided in Table S10.

To better understand the impact of these migrations, we identified haplogroups associated with subsistence strategies shared by populations from EA and MSEA, using both spatiotemporal data and prior research (Methods). Among the lineages, C2b1a, C2b1b1, D1a1a1a1, D1a1b1a, N1b2, O1a1a2, O1b1a2, O2a1b1a1a1a1, O2a2b1a1a, O2a2b2, and Q1a1a were classified as lineages associated with millet‐farming archaeological contexts, whereas O1a1a1, O1b1a1a1a1, O1b1a1a1b, O2a1b1a2a, O2a2a1a2a1, and O2a2b1a2a were classified as lineages associated with rice‐farming archaeological contexts (Table S10). Northern millet‐farming‐related lineages were widespread among Chinese populations, with northern Han individuals exhibiting an average frequency of 49.73% (Figure 7A; Table S10). In contrast, rice‐farming‐related lineages predominated in southern China. Additionally, lineages associated with Siberian foraging archaeological contexts significantly contributed to the genetic makeup of northern and southwestern Chinese groups, whereas those lineages associated with western Eurasian pastoralism archaeological contexts had a more limited presence. As anticipated, rice‐farming‐related haplogroups were the most common in MSEA (32/37, >25%). Notably, some groups displayed elevated frequencies of northern millet‐farming‐related lineages, such as the Sila, Hanhi, and Lachi from Vietnam (>69%); and the Karen, Shan, and Nyahkur from Thailand (>50%). Correlation analyses revealed significant geographic distribution patterns: millet‐farming‐associated haplogroups showed positive correlations with latitude, whereas rice‐farming‐associated lineages showed negative correlations (Figure 7C). Meanwhile, foraging‐ and pastoralism‐related lineages also showed significant correlations with geographic coordinates, but in opposite directions: the former was positively associated with longitude, while the latter was negatively associated. However, such correlations reflect geographic associations rather than causal relationships. After integrating all available data into eight meta‐populations based on haplogroup assignments and geographic location, the genetic contributions of Neolithic farmers to EA became more pronounced (Figure 7B; Table S10). Northern Han and ethnic minorities predominantly carried lineages associated with millet farmers, while also showing notable influences from northward‐expanding rice farmers and other hunter‐gatherers. In contrast, southern Han and ethnic minorities largely inherited rice‐farming lineages but appeared to have also been admixed with southward‐expanding millet farmers. These Neolithic farming groups further shaped the genetic landscape of ancient and present‐day MSEA populations. These findings suggest that the initial southward migration of northern millet‐farming populations influenced the paternal genetic structure of southern East Asians, followed by further southward movements of both northern and southern farmers, which contributed to the current paternal genetic landscape of MSEA groups.

## 3 Discussion

### 3.1 Paternal Evidence for Population Communication between Northern and Southern East Asians

#### 3.1.1 Demic Diffusion of Han Culture

Human paternal genomic resources from ethnolinguistically diverse East and Southeast Asian populations were severely underrepresented in human genome research [2, 45, 60]. Here, we reconstruct the paternal genetic history of EA and MSEA using a dataset that includes 584 whole Y‐chromosome sequences, 7877 high‐density genotyping profiles, and 5974 modern [35, 44, 58, 59, 60, 61, 62, 66] and ancient Eurasian reference genomes (Table S2) [67]. This integrative dataset fills a critical sampling gap in southwest China and reveals detailed patterns of paternal genomic diversity, origin, migration, and admixture. We reconstructed a high‐resolution Y‐chromosome phylogeny across ethnolinguistically diverse East and Southeast Asian populations and explored their genetic affinity using multiple statistical methods, revealing fine‐scale paternal genetic structure across these populations. We also reported a fully resolved ML tree incorporating ancient eastern Eurasian genomes, providing refined resolution of deep phylogenetic relationships between East and Southeast Asian farmers.

The expansion of Han culture has sparked a lengthy debate between the demic diffusion and cultural diffusion models [11, 68]. Historical records document multiple waves of Han‐related southward migration, driven by wars and famines in the north [13, 19]. Three major migrations, including the Yongjia Rebellion (Western Jin Dynasty, AD 265–316), the An‐Shi Rebellion (Tang Dynasty, AD 618–907), and the Jingkang Incident (Southern Song Dynasty, AD 1127–1279), involved over 6 million immigrants. Smaller‐scale migrations have also occurred continuously over the past two millennia. These historical accounts strongly support the demic diffusion model, which our genetic findings corroborate. Our results showed higher genetic diversity in Han populations, particularly northern Han, than in minority groups, suggesting deeper ancestral roots and greater admixture in northern Han populations. This contrasts with previous mtDNA studies [18, 69], suggesting greater diversity in southern Han populations. Contrasting patterns of uniparental diversity likely reflect sex‐biased demographic processes. Higher Y‐chromosomal diversity in northern Han populations may result from repeated male‐driven expansions and large effective population sizes in the Yellow River basin. In contrast, successive founder effects during southward migrations reduced paternal diversity in southern Han groups. PCA across East Asians revealed that southern Han populations clustered more closely with northern Han populations than with southern minority groups (Figure S6), a trend that becomes even more pronounced at the broader Eurasian scale (Figure 3B). Additionally, *F*st values between northern and southern Han populations were low, substantially lower than those observed between Han and most minority groups (Table S5). AMOVA analysis showed minimal genetic differentiation between northern and southern Han populations (0.97%), consistent with mtDNA findings [70]. Despite slight genetic differentiation between the northern and southern Han populations at the haplogroup level (Table S8), shared upstream haplogroups (e.g., O2a1b, O2a2b) underscored their paternal homogeneity (Figure 4A). Moreover, the spatiotemporal distributions of aDNA and haplogroups related to subsistence strategies (Figures 5 and 6) highlighted the role of millet‐farming migrations and interactions with southern rice‐farming groups in shaping the Han's genetic landscape [20]. These findings demonstrate that population expansion has driven significant migration and admixture, facilitating the spread of Han culture, and are consistent with the reconstructed dispersal and divergence patterns of the Tai‐Kadai language phylogeny from China and MSEA [71].

#### 3.1.2 The Role and Development of Rice Agriculturalists in Southern China

While northern China is recognized as the birthplace of millet farming and Han culture, southern China is considered the cradle of rice agriculture [72]. Over 10000 years ago, early farmers in the middle and lower Yangtze River region initiated rice domestication, laying the foundation for the development of South China's civilization [73]. These early rice farmers likely influenced several East and Southeast Asian populations, including Hmong‐Mien, Austroasiatic, Tai‐Kadai, and Austronesian groups [11, 22, 23, 74, 75]. Notably, recent ancient DNA evidence shows that Yangtze River rice farmers were genetically closely related to Neolithic to present‐day southeastern coastal and Austronesian‐speaking Chinese populations, indicating that the latter derived much of their ancestry from the Yangtze River basin and reflecting the profound demographic impact of rice agriculturalists in southern China [7]. However, the fine‐scale paternal genetic structure of southern Chinese populations remains underexplored. To address this gap, we included extensive sampling from minority groups in underrepresented southwestern and coastal regions and identified several O1/O2‐related sub‐lineages that served as the initial founding lineages of rice farmers and incoming southward millet farmer lineages. PCA and *F*st analyses revealed that linguistically diverse southern minorities tended to cluster together (Figure 3B,C), suggesting a strong correlation between Y‐chromosomal variation and linguistic affiliation. Among these groups, the Tibeto‐Burman and Hmong‐Mien‐speaking populations showed closer genetic relationships with Han populations, suggesting possible historical genetic interactions. In contrast, more isolated groups, such as the Yao and Maonan from Guizhou and Tibetans, appeared more genetically distinct, likely due to geographical isolation or unique origin. AMOVA results confirmed genetic heterogeneity within southern minorities (Table 2). Negative Tajima's *D* and Fu's FS indicate recent paternal expansion in South China. Our analyses identified two key founding paternal lineages: O1b1a1a1a1a1a‐F2758 (Tai‐Kadai) and O2a2a1a2a1a1‐F1275 (Hmong‐Mien) (Figure 4). Haplogroup patterns related to subsistence strategies further revealed a greater influence of northern millet farmers on southwestern minorities and a more substantial presence of rice‐farming lineages among coastal groups, in agreement with recent ancient genomic evidence (Figure 6) [7]. Highland minorities, such as Tibetans, showed genetic influences from both millet farming and other complex genetic flows. These findings suggest that southern Chinese minorities can be broadly categorized into three genetic clusters: southern coastal minorities (Tai‐Kadai speakers), southwestern minorities (Hmong‐Mien speakers), and highland minorities (Tibeto‐Burman speakers).

### 3.2 Paternal Genetic Connection between Neolithic East Asian Farmers and Southeast Asian Populations

Genetic evidence from aDNA [8, 9, 10, 76], archaeology [76, 77], linguistics [71], autosomal DNA [29, 78, 79], and mtDNA [80, 81, 82] suggests that two major agricultural expansions from EA significantly shaped the genetic structure of Southeast Asian populations during and after the Neolithic period. These expansions influenced the Tai‐Kadai, Hmong‐Mien, Austroasiatic, and Austronesian peoples, raising an important question: which paternal lineages contributed to these genetic contributions, and to what extent? Our study, incorporating newly sampled Tai‐Kadai, Hmong‐Mien, and Sino‐Tibetan populations, along with Eurasian reference data, provides a robust framework to address this question. The results offer compelling evidence supporting the direct paternal genetic connections between ancient Chinese farmers and modern populations from MSEA. Despite lower coverage of MSEA data, shared haplogroups across 17 clades indicate significant genetic continuity, with haplogroup O reflecting Upper Pleistocene and Neolithic genetic exchanges (Figures 1 and 2). The concentration of expansion events since five kya may suggest a potential impact of Neolithic agricultural expansion [83, 84]. The sharp increase in lineage expansion around 3.5 kya further aligns with population growth driven by improvements in agricultural production systems, increased social complexity, and state formation during the Bronze Age [85, 86]. Phylogeographic analyses revealed widespread lineage radiations, with north‐to‐south dispersals predominating. Several clades exhibited regional specificity, demonstrating both shared ancestry and localized differentiation across EA and MSEA. We note that the lower‐coverage MSEA samples contain fewer informative SNPs, which may, in some cases, bias placements toward upstream nodes and reduce phylogenetic resolution at the terminal level. Accordingly, we focus our interpretations on robust, clade‐level trends rather than fine‐scale terminal substructure. Notably, the consistent phylogenetic patterns across datasets, together with concordant evidence from multiple analytical approaches, support the reliability of our evolutionary inferences. For example, genotyped data further highlighted striking genetic similarities between East and Southeast Asian groups, especially among Tai‐Kadai and Hmong‐Mien‐speaking populations (Figure 3), corroborating findings from recent linguistic and genetic studies [29, 71, 80, 87]. Haplogroup distributions linked to subsistence strategies showed that populations from MSEA have been significantly influenced by paternal lineages related to southern rice farmers (Figure 6). However, northern millet farmers also contributed substantially to the genetic makeup of several Southeast Asian groups. These findings underscore the dual influence of Neolithic southern rice farmers and northern millet farmers in shaping the paternal genetic legacy of MSEA populations. Although we incorporate extensive Y‐chromosome data from southern Chinese ethnic minorities, which helps mitigate sampling gaps among multi‐ethnic populations, further inclusion of broader whole Y‐chromosome sequences remains essential to address the current bias toward Han populations. In addition, integrating more mtDNA data and additional aDNA samples from southern EA will be critical to test for potential sex‐biased Neolithic expansions and to refine the timeline of these genetic exchanges.

## 4 Conclusion

This study examined MSY variation in 14435 individuals, primarily from eastern Eurasia, with a focus on EA and MSEA. By reconstructing a high‐resolution Y‐chromosome phylogeny of modern eastern Eurasians with calibrated dates, we provide new insights into the deep evolutionary history of eastern Eurasian populations. We also present the highest‐resolution Y‐chromosome phylogeny to date for ancient East and Southeast Asians, enabling a detailed exploration of the paternal connections between Neolithic Chinese farmers and modern and ancient MSEA populations. Our results identified fine‐scale paternal genetic structures within China and emphasized the demic diffusion model of Han culture and Neolithic agricultural technologies. This study suggests that the genetic landscape of southern China was significantly influenced by the southward migration of northern East Asian populations, followed by further interactions and migrations between northern and southern Neolithic farmers, which shaped the paternal composition of MSEA.

## 5 Methods

### 5.1 Sample Collection

Saliva samples were collected from 8461 unrelated individuals with their informed consent. DNA was extracted from 584 males used for whole‐genome sequencing and 7877 males from 34 provinces for genome‐wide genotyping. A QIAamp DNA Mini Kit (QIAGEN, North Rhine‐Westphalia, Germany) was used to extract and purify the DNA. The DNA concentrations were subsequently quantified on a Qubit 3.0 fluorometer via the Qubit dsDNA HS Assay Kit (Thermo Fisher Scientific, Waltham, USA) according to the manufacturer's instructions. Ethical approval for this study was granted by the Medical Ethics Committee of West China Hospital of Sichuan University (2023‐306), and the study was conducted in accordance with the 2013 Helsinki Declaration [88]. All procedures for sample collection and experiments in this study followed the recommendations of the Human Genetic Resources Administration of China (registration no. 2025BAT00892).

### 5.2 Whole‐Genome Sequencing, Genotyping, and Quality Control

All newly reported Y‐chromosome sequences were generated on the Salus Pro genetic sequencer (Shenzhen Salus BioMed Co., Ltd.) with an average coverage of 87×. FASTQ files were aligned to the human reference genome GRCh37 using BWA‐MEM v0.7.13 [89]. Read duplicates were removed with Picard v3.0.0 (https://broadinstitute.github.io/picard/), and base quality scores were recalibrated using GATK v4.2.6.1 [90]. Variant calling was conducted with GATK HaplotypeCaller [91]. Newly sequenced samples were merged with globally published datasets using the GATK CombineVariants and GenotypeGVCFs modules [92]. To account for coverage variation among samples, two datasets were constructed: Dataset 1 includes 3618 new and reference samples with ca. 8.4 Mb of Y‐chromosome data per sample, excluding 1748 MSEA individuals with only ca. 2.3 Mb genomic coverage. All‐sites VCF files were filtered with bcftools v1.8 [91] by excluding those with > 5% missing calls, base quality < 30, or heterogeneity > 15%. After filtering, Dataset 1 retained 104540 high‐quality SNPs. Dataset 2 comprises all 5366 whole‐genome sequences. The 2.3 Mb sequences, however, were integrated with the other 8.4 Mb sequences through a placement approach, as described in the phylogenetic inference section below. Detailed information for all samples is provided in Table S1. For the chip data from 2636 males, we genotyped 11457 SNPs using the Illumina microarray platform for the final analysis. Individuals with familial relationships were filtered out. Finally, 2206 unrelated individuals within each ethnolinguistic group with sample sizes greater than 20 were considered in the subsequent analysis. These individuals from 31 ethnolinguistic populations belonging to the Hmong‐Mien (Miao, Nanjing, She, and Yao), Mongolic (Mongolian), Sinitic (Han, Hui, and Tanka), Tai‐Kadai (Bouyei, Dong, Li, Maonan, and Zhuang), Tibeto‐Burman (Bai, Qiang, Tibetan, and Tujia), and Tungusic (Manchu) language families.

### 5.3 Phylogenetic Inference and Time Estimation

We merged our data with publicly available modern and ancient genomes via bioinformatics pipelines and obtained the final high‐coverage and low‐coverage Y‐chromosomal dataset for phylogeny reconstruction (Tables S1 and S2) [35, 44, 58, 59, 60, 61, 62, 66, 67]. For the 3618 samples with ca. 8.4 Mb of Y‐chromosome data, an ML phylogenetic tree was constructed using RAxML‐NG v1.2.2 [93] with the GTR+G+I model. This involved 200 rapid bootstrap runs followed by a thorough ML search. One sample belonging to haplogroup NO and two samples exhibiting excessively long branch lengths were removed. The ca. 2.3 Mb MSEA samples and published aDNA data (Tables S1 and S2) were subsequently placed onto this ML tree using pathPhynder [64]. Some males lack BAM files and are available only as haplogroup genotype results. Both resulting trees were visualized using iTOL [94]. Phylogenetic inference and coalescence time estimation were further performed on a dataset of 823 sequences from dataset 1 (including 584 newly generated samples and some references) using BEAST v2.7.6 [95]. Two parallel runs were conducted with different random seeds, using identical parameters: a Bayesian skyline coalescent tree prior, a general time‐reversible substitution model with a gamma‐distributed rate, and a strict molecular clock with 10 groups. Coalescence times were calibrated based on published divergence dates and substitution rates. The calibration point for node NO‐F549 was set at 41900 years with a standard deviation of ±1708 years [96]. The substitution rate was 7.6 × 10^−10^ mutations per site per year (95% confidence interval: 6.7 × 10^−10^ to 8.6 × 10^−10^). Each run consisted of 140 million chains, with logging every 4000 steps. Outputs from both runs were combined with LogCombiner, excluding the initial 25% as burn‐in. Effective sample sizes (ESS > 200) were assessed and kept using Tracer v1.7.2 [97]. The maximum clade credibility tree was then visualized in FigTree and iTOL. As this tree file contained node information, we used R packages to generate plots of new node accumulation and total node counts over time. We report phylogenetic time estimates in kya (thousand years ago), whereas archaeological dates for ancient individuals follow the conventional radiocarbon‐based BP (Before Present, 1950) format. These two time scales are kept separate because of their distinct reference systems.

### 5.4 Haplogroup Classification and Genetic Diversity

HaploGrouper was used to classify each sample's haplogroup [98]. Microsoft Excel was used to analyze haplogroup distributions by ethnicity, language, and geographic region. Mann‐Whitney U tests and Welch's t‐tests were conducted in Python using the SciPy package to assess whether haplogroup frequencies differed significantly across geographically distinct populations [99]. The symbol ^*^ indicates 0.01 ≤ *p* < 0.05, ^**^ indicates 0.001 ≤ *p* < 0.01, and ^***^ indicates *p* < 0.001. Arlequin v3.5 software was used to calculate Pi, Theta(S), Tajima's *D*, and Fu's FS [100]. DnaSP 6 was used to estimate HD, and HGD was calculated via Microsoft Excel [101]. All the analysis results above were visualized via the matplotlib package in Python [102]. Pi represents the mean number of differences between all pairs of haplotypes within a population and serves as an indicator of genetic diversity. Theta(S) is an estimate based on the number of segregating sites (S) and reflects the average mutation rate per site across DNA sequences in a population. HGD refers to the probability that two randomly chosen sequences from a single population belong to different haplogroups, whereas HD denotes the probability that two randomly chosen sequences belong to different haplotypes. Both HD and HGD measure the degree of genetic variation within a population. Tajima's *D* test and Fu's FS are used to examine whether DNA sequences conform to expectations of neutral evolution.

### 5.5 Genetic Relationships and Demographic History Reconstruction

PCA based on fourth‐level haplogroup frequencies was performed using Y‐LineageTracker [102] and the Python sklearn package [103]. Similarly, Pearson's correlation analysis was conducted in Python via the SciPy package to investigate whether the PC values were correlated with latitude and longitude [99]. Pairwise *F*st and AMOVA were computed via Arlequin v3.5 [100]. Significance was assessed using permutation tests implemented in Arlequin. The *F*st values for the East Asian populations studied were calculated from sequences, whereas the *F*st values at the Pan‐Asian scale were computed from haplogroup frequencies at level 4. The phylogenetic relationships were further delineated using a UPGMA tree based on the *F*st genetic distance matrix, constructed in MEGA v12.0 [104]. To elucidate the genetic relationships among populations, MJ networks were constructed and visualized via the FastHaN program [105]. HDHs were identified using a differentiation frequency threshold of 0.05, meaning that haplogroups with frequency differences of 0.05 or more between populations were initially considered potential HDHs. We then analyzed the distribution of these haplogroups across populations using chi‐square and Fisher's exact tests in Python using the SciPy package. Only haplogroups that showed statistical significance in both tests were classified as real HDHs. The PCA and *F*st outputs were visualized in R. The UPGMA and ML trees were then modified in iTOL. The HDHs were visualized in Python via the matplotlib package. Pearson's correlation coefficients were calculated in Python using the SciPy package to explore relationships between haplogroup frequencies and geographic coordinates (longitude and latitude).

### 5.6 Spatiotemporal Statistics

Y‐LineageTracker was used to calculate the haplogroup frequency of one province‐defined population at different levels of the terminal haplogroup [106]. ArcGIS Pro was used to visualize the geographic distributions of sample resources and the spatiotemporal distribution of aDNA, perform optimized hotspot analysis, and infer the potential phylogeographic origins of the paternal founding lineages (https://www.esri.com/en‐us/arcgis/products/arcgis‐pro/overview).

### 5.7 Definitions of Haplogroups Linked to Subsistence Strategies

We first identified 17 haplogroups shared between present‐day populations from EA and MSEA and five region‐specific lineages based on the ML tree. A haplogroup was considered shared between the two regions only if it reached a particular frequency in both and included more than 30 downstream individuals. We subsequently matched the aDNA samples to specific haplogroups using pathPhynder and combined the estimated divergence and expansion times to define the possible founder populations for each younger paternal lineage. By observing the temporal and spatial distribution maps of ancient samples, potential migration routes of ancestry were inferred from the distributions of the oldest to progressively more recent samples. When aDNA evidence was inadequate, haplogroup origins were inferred from the ML tree topology in Figure 1, in combination with the geographic distribution of modern samples. Additionally, by integrating archaeologically defined cultural sequences, the geographic distribution of the oldest samples, and previous studies, we defined haplogroups associated with specific subsistence strategies and their geographic distribution. Notably, the currently available samples are limited. Future studies incorporating more widely distributed aDNA genomes are expected to enable more precise reconstructions of population history.

### 5.8 Statistical Analysis

We conducted all statistical analyses using Python (SciPy) and Arlequin v3.5, unless indicated otherwise. We did not exclude any data from the analyses unless explicitly noted. We assessed haplogroup frequency differences between populations using Mann‐Whitney U tests, Welch's t‐tests, chi‐square tests, or Fisher's exact tests, as appropriate. Correlations between genetic variables and geographic coordinates were evaluated using Pearson's correlation coefficient. AMOVA, Tajima's *D* test, Fu's FS, and pairwise *F*st were conducted in Arlequin. Statistical significance was set at *p* < 0.05 unless stated otherwise, with a threshold of *p* < 0.02 for Fu's FS.

## Author Contributions

Y.L., M.W., and G.H. contributed equally to this work. C.L., M.W., R.T., and G.H. conceived and designed the study. M.W. and G.H. developed the methodology. Y.L., L.L., M.Z., Z.W., Z.Z., Y.W., and L.Z. conducted the investigation. Y.L., L.L., T.Y., L.L., Y.J., and Y.F. prepared the visualizations. M.W., C.L., R.T., and G.H. supervised the project. Y.L., M.W., and G.H. wrote the original draft. B.Z., M.W., and G.H. reviewed and edited the manuscript.

## Conflicts of Interest

The authors declare no conflicts of interest.

## Supporting information




**Supporting File 1**: advs73784‐sup‐0001‐SuppMat.pdf.

**Supporting File 2**: advs73784‐sup‐0002‐Supp Tables.xlsx.

## Data Availability

The data that support the findings of this study are available in the supplementary material of this article.
