# Supplementary material for: Modern and Ancient Genomes Reveal Neolithic Paternal Expansions of Millet and Rice Farmers and Demic Diffusion from China into Mainland Southeast Asia
Source: Adv Sci (Weinh). 2026 Jan 21;13(17):e15930. doi: 10.1002/advs.202515930 (PMC13042612; doi:10.1002/advs.202515930)
Supplement: Supplementary file 1 — Supporting File 1: advs73784‐sup‐0001‐SuppMat.pdf. [file ADVS-13-e15930-s001.pdf]

Supporting Information

Modern and Ancient Genomes Reveal Neolithic Paternal Expansions of Millet and Rice Farmers and Demic Diffusion from China into Mainland Southeast Asia

Yunhui Liu, Lintao Luo, Yutong Jiang, Minzhu Zhao, Ting Yang, Zhiyong Wang, Lisiteng Luo, Yuhang Feng, Zihao Zhu, Yuzhu Wang, Limei Zhang, Bofeng Zhu, Chao Liu, Renkuan Tang*, Mengge Wang*, Guanglin He**

Supplementary Figures:

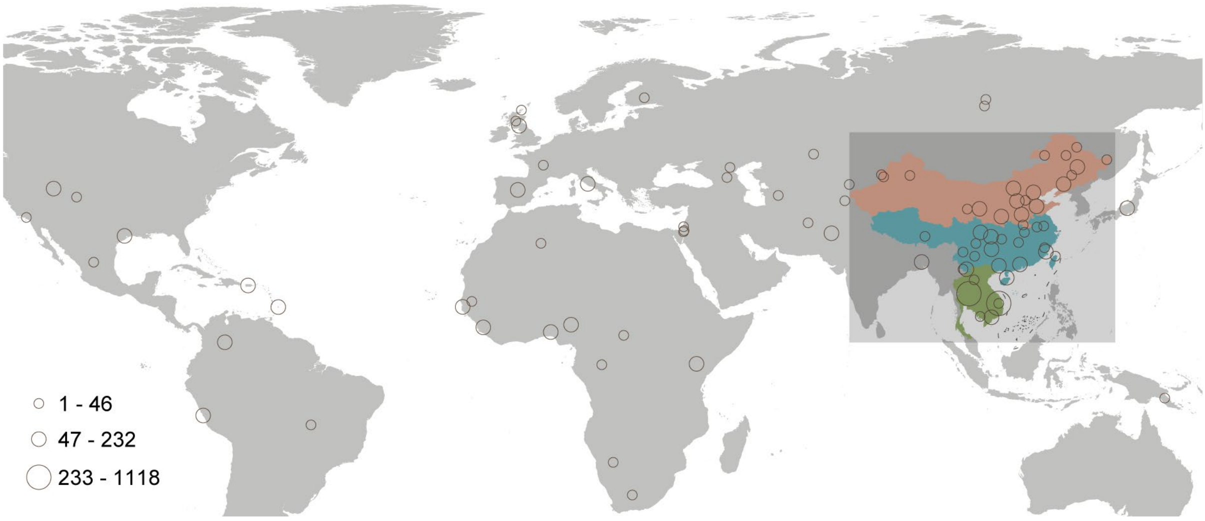

Figure S1. Geographic distribution of present-day males in this study. Data types comprise both WGS and high-density genotyping. The size of each brown circle is proportional to the sample count at each location.

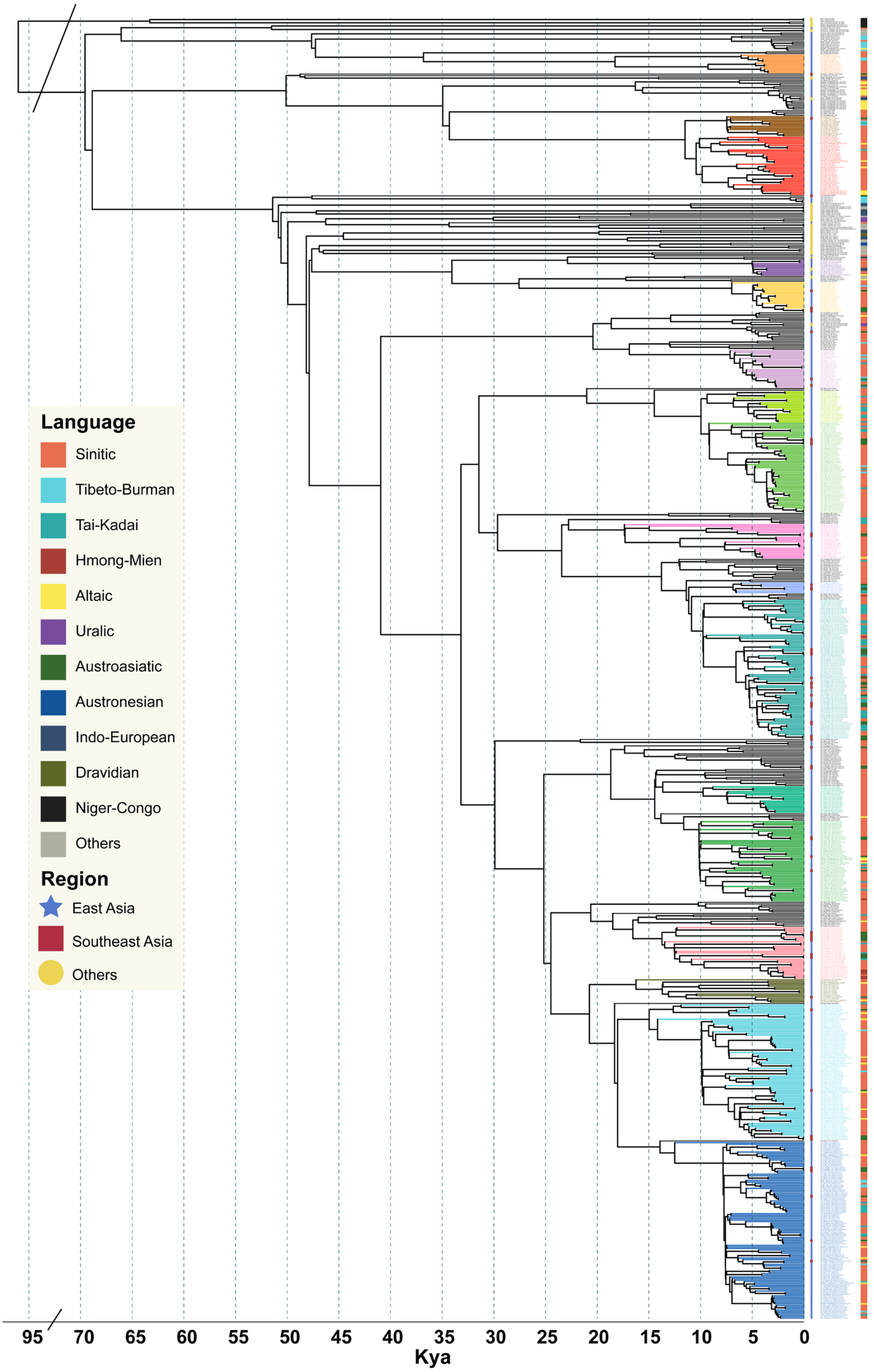

Figure S2. Time-calibrated phylogenetic tree with sample labels, corresponding to Figure 2A. This tree is identical to Figure 2A but includes sample labels. For the 17 clades shared between EA and MSEA populations, label colors correspond to the branch colors of their respective lineages.

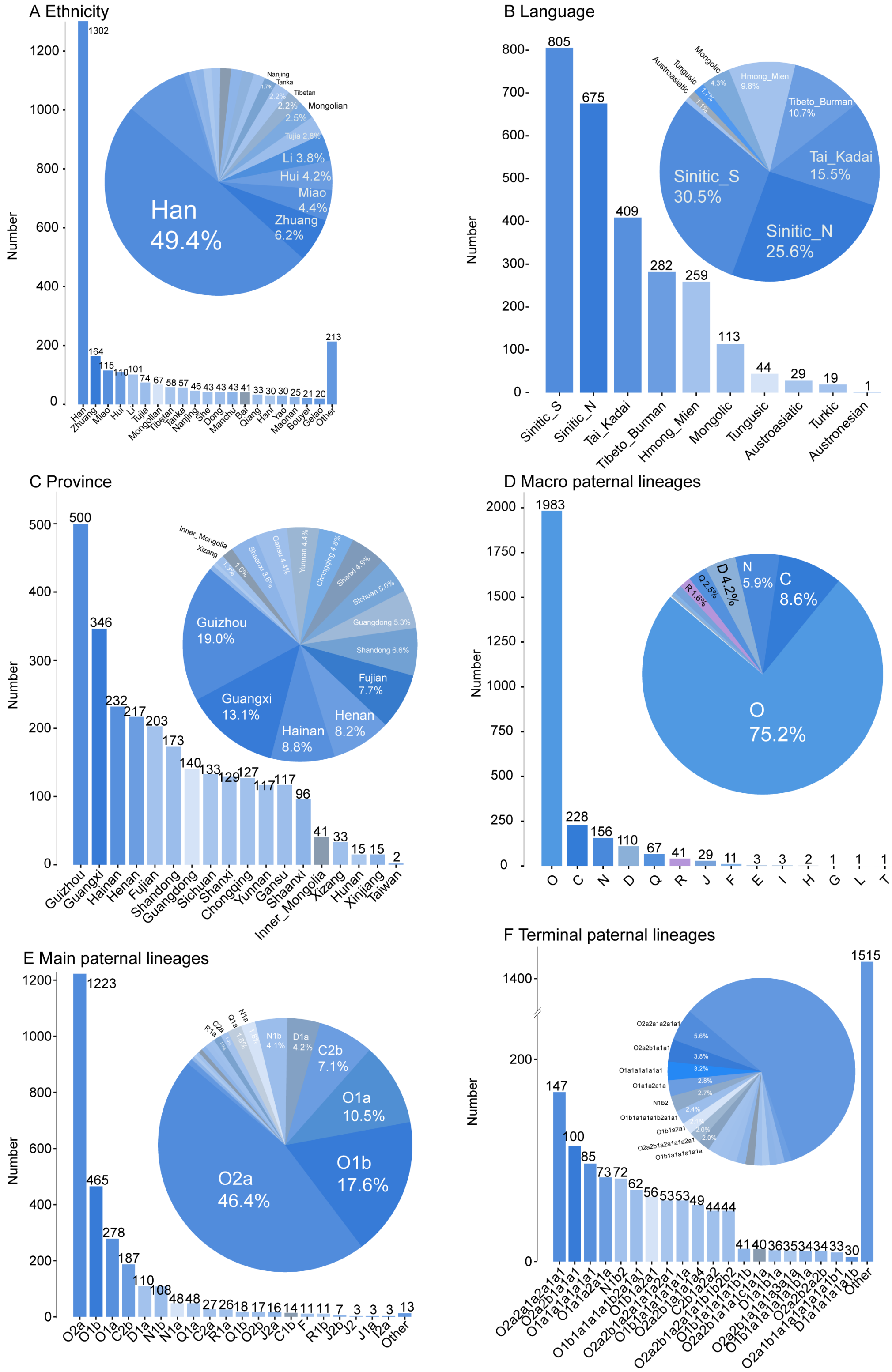

Figure S3. Geographic distribution and associated information of 2,636 newly reported genotyped samples. (A) Ethnic groups; (B) Linguistic families; (C) Provinces; (D) Macro paternal lineages; (E) Main paternal lineages; (F) Terminal paternal lineages.

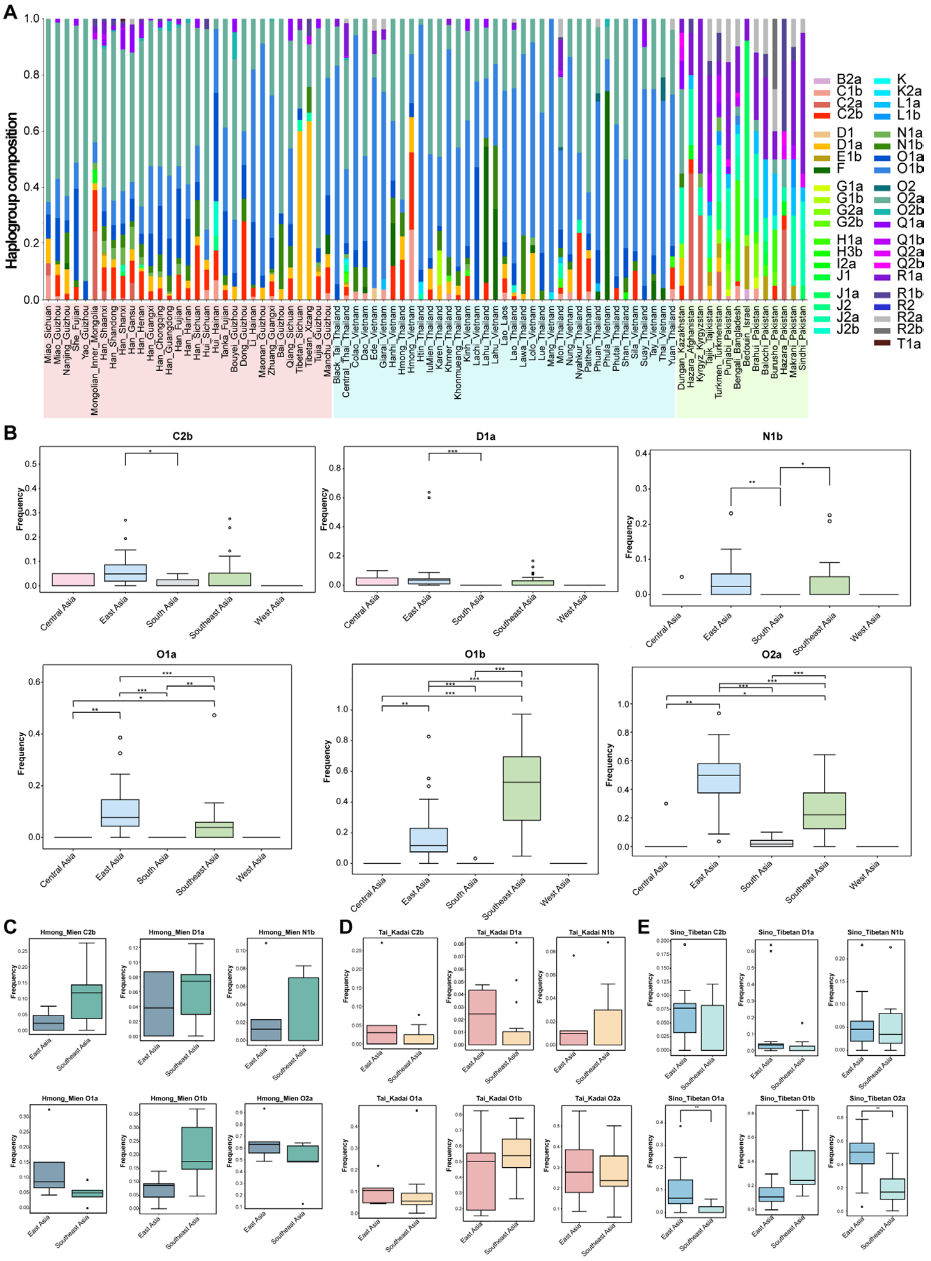

Figure S4. Level 3 paternal haplogroup composition and statistical tests. (A) Distribution of level 3 haplogroups across studied and reference populations. Background colors denote geographic origin: red for East Asia, teal for Southeast Asia, and green for West, South, and Central Asia. (B–E) Statistical comparisons of haplogroup frequencies using Mann–Whitney U and Welch's t-tests. Each data point represents the haplogroup frequency of one population. The symbol * indicates $0.01 \leqslant p < 0.05$, ** indicates $0.001 \leqslant p < 0.01$, and *** indicates $p < 0.001$. (B) Across major Asian regions (Central Asia, n = 5; East Asia, n = 31; South Asia, n = 8; Southeast Asia, n = 37; West Asia, n = 1). (C) Among Hmong-Mien populations in East Asia (n = 5) and Southeast Asia (n = 5). (D) Among Tai-Kadai populations in East Asia (n = 5) and Southeast Asia (n = 15). (E) Among Sino-Tibetan populations in East Asia (n = 5) and Southeast Asia (n = 7).

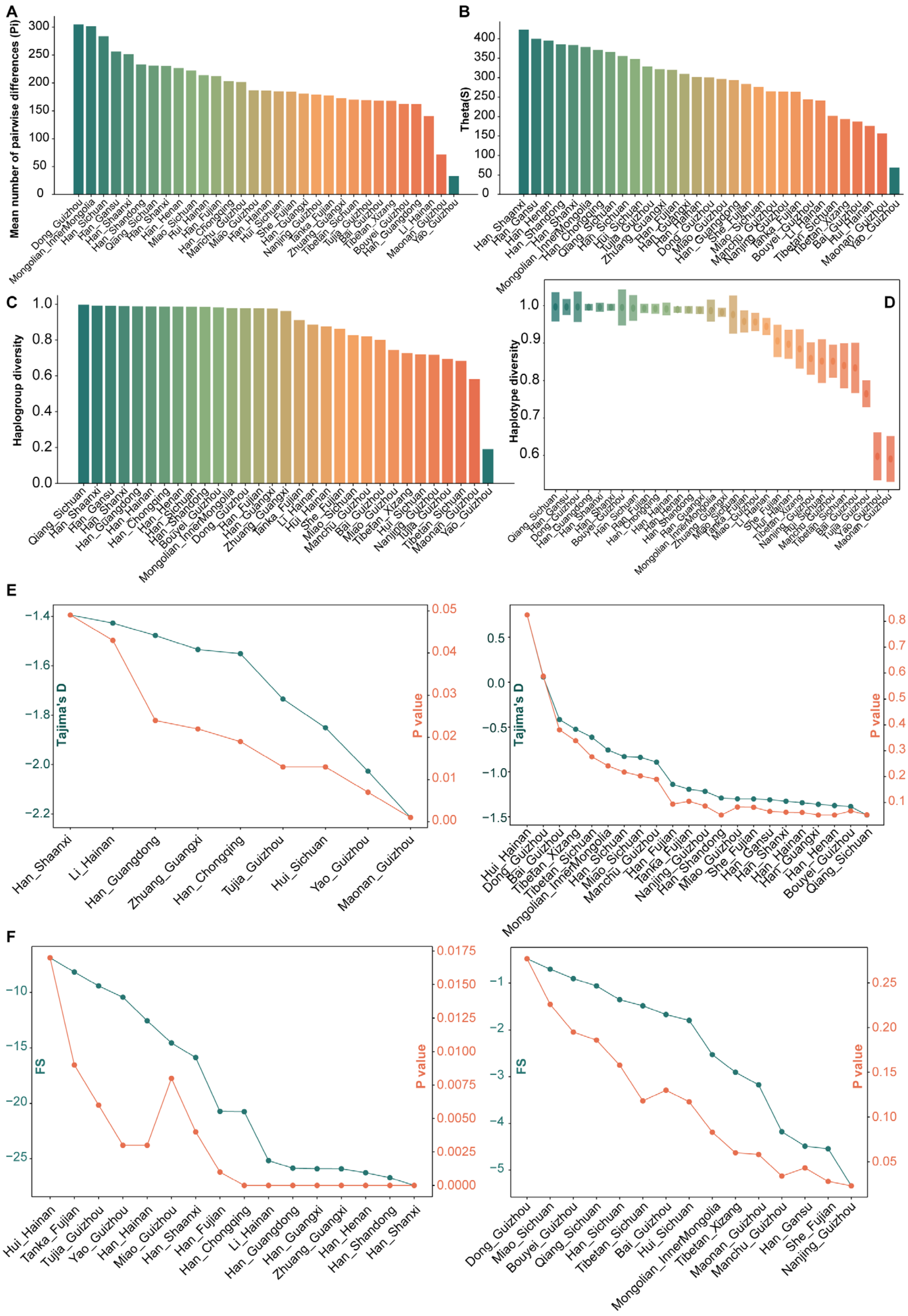

Figure S5. Genetic diversity metrics for 31 newly reported populations in China. (A) Mean pairwise differences (Pi); (B) Theta (S); (C) Haplogroup diversity (HGD); (D) Haplotype diversity (HD); (E) Tajima's *D*; (F) Fu's Fs. In (E) and (F), the left panels show populations with statistically significant negative values ($p < 0.05$ for Tajima's D and $p < 0.02$ for Fu's Fs), while the right panels show populations with non-significant values. Y-axis labels are color-coded to match the corresponding data series. For example, in (E), the left Y-axis refers to Tajima's *D* (green curve), and the right Y-axis refers to *p* values (orange curve).

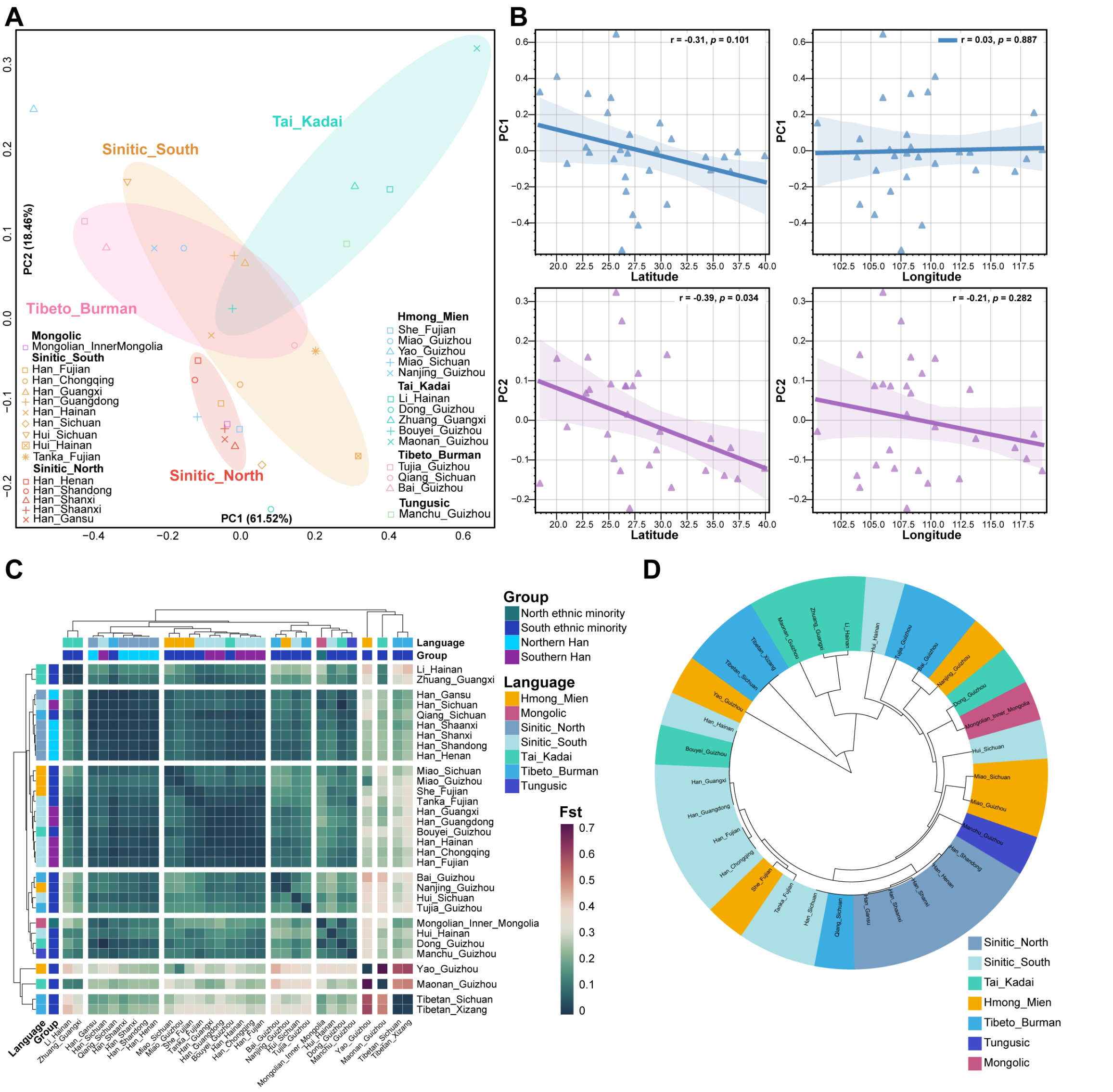

Figure S6. Population genetic structure of 31 newly reported Chinese populations. (A) Principal component analysis (PCA) of 29 populations. Two populations were removed as outliers. (B) Pearson correlation between PC values and geographic coordinates (latitude and longitude) of the corresponding populations (n = 29), and $p < 0.05$ was considered significant. (C) Pairwise genetic distance (*F*st) analysis. Darker green indicates lower *F*st values (closer genetic affinity), while deeper orange represents higher *F*st values (greater genetic divergence). (D) UPGMA phylogenetic tree based on *F*st values.

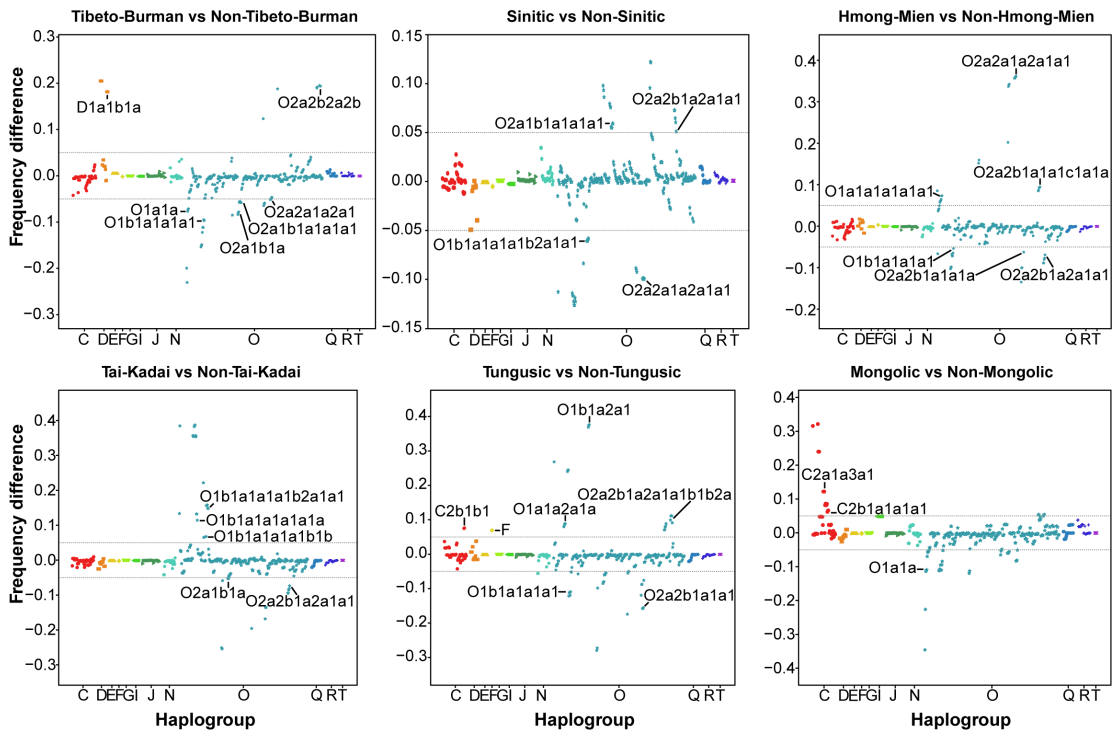

Figure S7. Highly differentiated haplogroups (HDHs) among populations of different language families. Only terminal subclades are labeled. A complete list of HDHs is provided in Table S9. Haplogroup distributions were compared using chi-square tests and Fisher's exact tests, as appropriate, with $p < 0.05$ considered statistically significant. Sample sizes (numbers of individuals per haplogroup and population) are shown in Table S9.

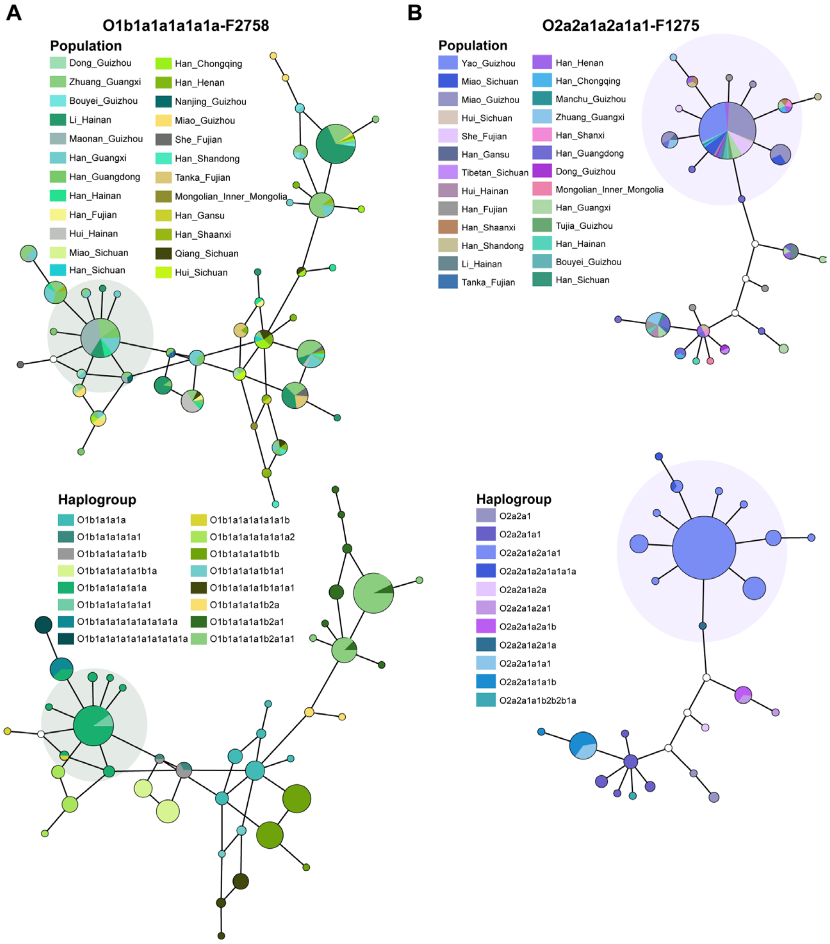

Figure S8. Median-joining network topologies based on Y-chromosome sequence data. Light green and light purple backgrounds highlight star-like phylogenies described in the main text. (A) Haplogroup O1b1a1a1a1a1a-F2758 among Tai-Kadai populations. (B) Haplogroup O2a2a1a2a1a1-F1275 among Hmong-Mien speakers.

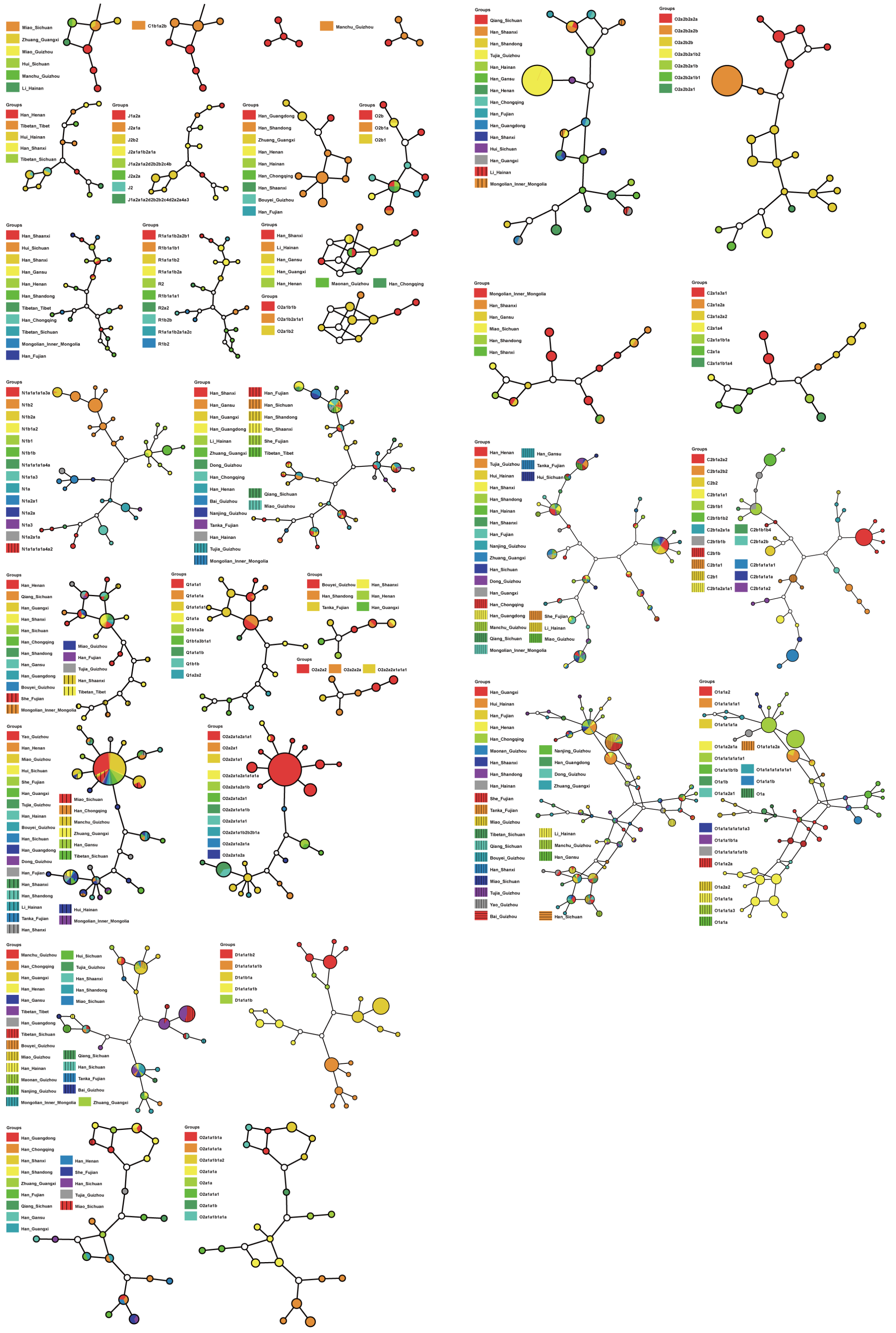

Figure S9. Median-joining networks based on sequence.

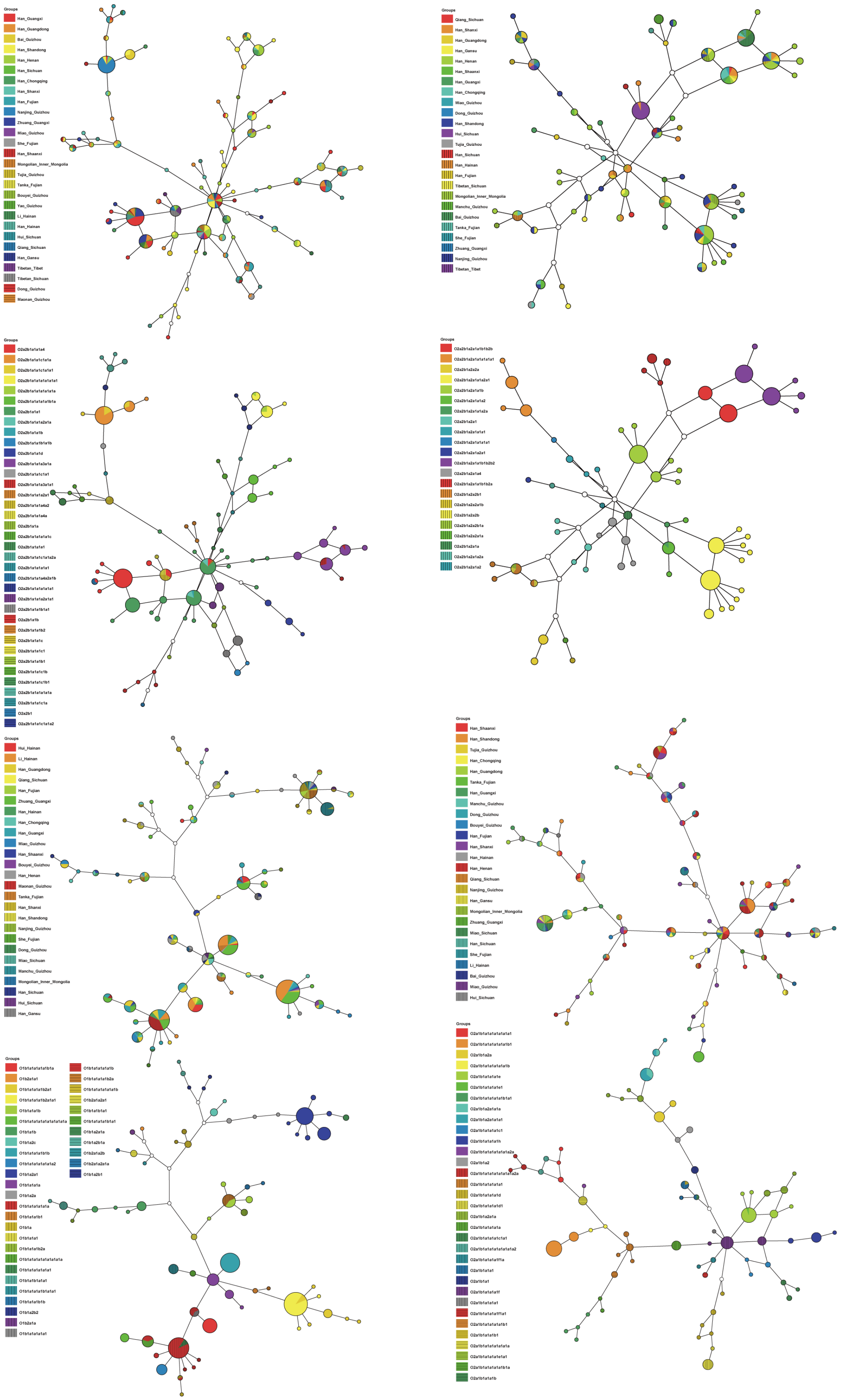

Figure S9. Median-joining networks based on sequence (continued).

Supplementary Tables:

Table S1. Data sources of modern population samples used in this study.

Table S2. Data sources of ancient population samples used in this study.

Table S3. Distribution of paternal haplogroup frequencies across different populations, with haplogroups unified to the second-level classification.

Table S4. Population diversity parameters across different groups.

Table S5. The *F*st value matrix (upper right triangle) and the corresponding p-values (lower left triangle) for the study populations.

Table S6. The *F*st value matrix (upper right triangle) and the corresponding p-values (lower left triangle) for the 82 studied and reference populations.

Table S7. AMOVA results based on different groups among East Asian.

Table S8. Highly differentiated haplogroups between pairwise comparisons of different populations. The second and third columns of each table represent the numbers of individuals carrying the corresponding haplogroup in each population.

Table S9. Highly differentiated haplogroups between pairwise comparisons of different populations (grouped by language family). The second and third columns of each table represent the numbers of individuals carrying the corresponding haplogroup in each population.

Table S10. The haplogroup composition associated with subsistence strategies in the 86 populations and meta-populations.
